# Downregulation of SOCS1 increases interferon-induced ISGylation during differentiation of induced-pluripotent stem cells to hepatocytes

**DOI:** 10.1016/j.jhepr.2022.100592

**Published:** 2022-09-23

**Authors:** Jasmine S. Edwards, Stephanie A. Delabat, Alejandro D. Badilla, Robert C. DiCaprio, Jinhee Hyun, Robert A. Burgess, Tiago Silva, Derek M. Dykxhoorn, Steven Xi Chen, Lily Wang, Yuji Ishida, Takeshi Saito, Emmanuel Thomas

**Affiliations:** 1 University of Miami Miller School of Medicine Department of Microbiology and Immunology, USA; 2 University of Miami Miller School of Medicine Department of Pathology, USA; 3 University of Miami Miller School of Medicine Department of Human Genetics, USA; 4 University of Miami Department of Public Health Sciences, USA; 5 Department of Medicine, Division of Gastrointestinal and Liver Diseases, Keck School of Medicine, University of Southern California, Los Angeles, California, USA; 6 Research & Development Department, PhoenixBio, Co., Ltd, Higashi-Hiroshima, Hiroshima, Japan; 7 USC Research Center for Liver Diseases, Los Angeles, California, USA

**Keywords:** HCV, Host Defense, Antiviral Response, Innate Immunity, ISG15, STAT1, SOCS1, Epigenetic Regulation, Liver Cancer, Hepatocellular Carcinoma, AFP, alpha-fetoprotein, ALB, albumin, FOXA2, forkhead Box A2, HB, hepatoblast, HCC, hepatocellular carcinoma, HLC, hepatocyte-like cell, IFN, interferon, iPSC, induced-pluripotent stem cell, IRF3, interferon regulatory factor 3, ISG, interferon-stimulated gene, JAK, Janus kinase, OCT4, octamer-binding transcription factor 4, PHHs, primary human hepatocytes, pSTAT1, phosphorylated STAT1, RIG-I, retinoic acid-inducible gene I, RLR, RIGI-like receptor, RNAseq, RNA sequencing, SOCS1, suppressor of cytokine signaling 1, STAT1, signal transducer and activator of transcription 1, TLR, toll-like receptor, UBE1L/UBA7, ubiquitin-activating enzyme E1, UbcH8/UBE2L6, ubiquitin-conjugating enzyme E2 L6, USP18, deconjugation enzyme ubiquitin specific peptidase 18

## Abstract

**Background & Aims:**

Increased expression of IFN-stimulated gene 15 (ISG15) and subsequently increased ISGylation are key factors in the host response to viral infection. In this study, we sought to characterize the expression of ISG15, ISGylation, and associated enzymes at each stage of differentiation from induced pluripotent stem cells (iPSCs) to hepatocytes.

**Methods:**

To study the regulation of ISGylation, we utilized patient samples and *in vitro* cell culture models including iPSCs, hepatocytes-like cells, immortalized cell lines, and primary human hepatocytes. Protein/mRNA expression were measured following treatment with poly(I:C), IFNα and HCV infection.

**Results:**

When compared to HLCs, we observed several novel aspects of the ISGylation pathway in iPSCs. These include a lower baseline expression of the ISGylation-activating enzyme, UBE1L, a lack of IFN-induced expression of the ISGylation-conjugation enzyme UBE2L6, an attenuated activation of the transcription factor STAT1 and constitutive expression of SOCS1. ISGylation was observed in iPSCs following downregulation of SOCS1, which facilitated STAT1 activation and subsequently increased expression of UBE2L6. Intriguingly, HCV permissive transformed hepatoma cell lines demonstrated higher intrinsic expression of SOCS1 and weaker ISGylation following IFN treatment. SOCS1 downregulation in HCV-infected Huh 7.5.1 cells led to increased ISGylation.

**Conclusions:**

Herein, we show that high basal levels of SOCS1 inhibit STAT1 activation and subsequently IFN-induced UBE2L6 and ISGylation in iPSCs. Furthermore, as iPSCs differentiate into hepatocytes, epigenetic mechanisms regulate ISGylation by modifying UBE1L and SOCS1 expression levels. Overall, this study demonstrates that the development of cell-intrinsic innate immunity during the differentiation of iPSCs to hepatocytes provides insight into cell type-specific regulation of host defense responses and related oncogenic processes.

**Impact and implications:**

To elucidate the mechanism underlying regulation of ISGylation, a key process in the innate immune response, we studied changes in ISGylation-associated genes at the different stages of differentiation from iPSCs to hepatocytes. We found that high basal levels of SOCS1 inhibit STAT1 activation and subsequently IFN-induced UBE2L6 and ISGylation in iPSCs. Importantly, epigenetic regulation of SOCS1 and subsequently ISGylation may be important factors in the development of cell type-specific host defense responses in hepatocytes that should be considered when studying chronic infections and oncogenic processes in the liver.

## Highlights

• ISGylation is a robust host defense response following HCV infection in hepatocytes.
• Stem cells demonstrate undetectable ISGylation following interferon stimulation.
• High baseline expression of SOCS1 protein negatively regulates ISGylation in stem cells.
• *SOCS1* gene expression is controlled through epigenetic regulation during differentiation.
• Downregulating SOCS1 enhances IFN-induced ISGylation which could have implications in cancer development.

## Graphical abstract

**Figure:**
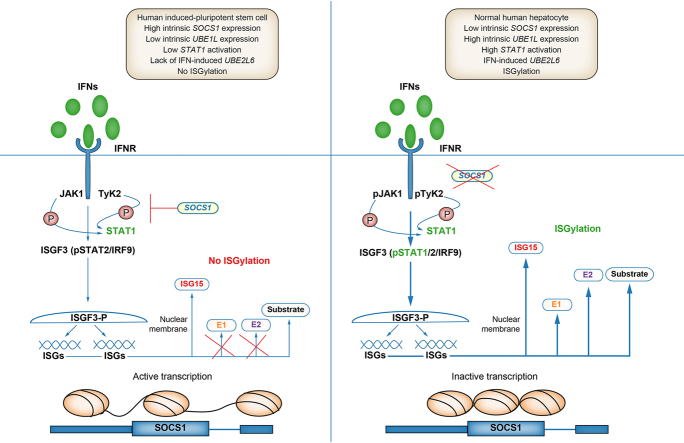

## Introduction

Very few viruses, of which HCV is one, can manifest as chronic pathogenic infections in humans.^1^^,^^2^ An estimated 58 million individuals are living with chronic HCV infection globally, and chronic infection with HCV is a major risk factor for the development of hepatocellular carcinoma (HCC), the third leading cause of cancer deaths worldwide according to the World Health Organization.^3^ The mechanisms underlying the establishment of chronic HCV infection in the liver are not fully characterized. The pertinent cell-intrinsic innate antiviral responses in hepatocytes have also not been fully elucidated.[4], [5], [6], [7], [8], [9], [10] A critical step in establishing an antiviral state is through the upregulation of interferon-stimulated genes (ISGs).^2^^,^^4^^,^^6^^,^[10], [11], [12], [13] Among these, interferon-stimulated gene 15 (ISG15) has been shown to have both antiviral and proviral activity against a variety of viruses including HCV.^14^^,^^15^

ISG15 is a 15 kDa protein upregulated following the activation of type I and III interferon signaling during HCV infection and it is expressed in many tissue types and cells throughout the human body, including the liver and hepatocyte.^16^ The cellular activities of ISG15 and associated proteins are involved in a ubiquitin-like post-translational process designated ISGylation. The specific proteins that catalyze ISGylation are the ubiquitin-activating enzyme E1 (UBA7/UBE1L), ubiquitin-conjugating enzyme E2 L6 (UbcH8/UBE2L6), three potential E3 ligases, and the deconjugation enzyme ubiquitin specific peptidase 18 (USP18).^17^ Unlike ubiquitination in which multiple ubiquitin proteins can be conjugated to a targeted protein, ISGylation involves only one ISG15 protein conjugated to a targeted protein. To date, around 300 proteins have been identified as ISGylation targets.^18^ Overall, ISG15 may regulate inflammatory responses through its conjugation to hundreds of host and viral proteins.[19], [20], [21] Importantly, recent studies have suggested that ISGylation is targeted to newly synthesized proteins^22^ during viral infection, implicating ISGylation of viral proteins as a general host defense mechanism. Importantly, ISG15 may be conjugated to HCV viral proteins to downregulate viral replication.^23^^,^^24^ However, most of these studies were performed in immortalized or transformed cell lines, which display blunted antiviral responses; as such, these studies require validation in primary cells that have fully intact innate antiviral responses. In addition, studying the development of these pathways that underlie host antiviral defense mechanisms are of paramount importance to further understand responses that may be tissue or cell type specific.

The reprogramming of adult somatic cells, such as fibroblasts, to generate induced-pluripotent stem cells (iPSCs) provides a newer model to study the development of cell-specific host defense mechanisms. Stem cells can self-renew with the capability of differentiating into any cell in the human body. The differentiation of iPSCs can be achieved using different combinations of small molecules and recombinant proteins.^25^ Importantly, published studies have begun to characterize the innate antiviral response within stem cells to viral pathogens. Chen *et al.*^26^ determined that stem cells express low levels of Toll-like (TLR) and RIG-I-like (RLR) receptors. Hong *et al.*^27^ demonstrated that iPSCs have an attenuated IFN response due to the expression of suppressor of cytokine signaling 1 (SOCS1), which inhibits signal transducer and activator of transcription 1 (STAT1) activation and subsequent transcription of ISGs. Wu *et al.*^28^ reported that despite an attenuated IFN response and low RLR and TLR expression, stem cells express a high level of a specific subset of intrinsic ISGs (attributed to epigenetic regulation) facilitating resistance to viral infection. It is clear that the basal expression of ISGs is dynamic, regulated during cellular differentiation, and may be controlled through epigenetic mechanisms that remain to be further clarified. Although ISG15 expression has previously been detected in stem cells and stem cell-derived cells, ISGylation and its subsequent regulation were not assessed in previous studies despite its important role in infection by HCV and other viruses.^20^^,^^21^^,^^23^

In this study, we sought to characterize the expression of ISG15, ISGylation, and associated enzymes at each stage of differentiation from iPSCs to hepatocytes. We report that ISGylation is inhibited in iPSCs following IFN treatment due to differential regulation of ISG15, UBE1L, and UBE2L6 through IFN-dependent and IFN-independent mechanisms associated with SOCS1 expression and epigenetic regulation, respectively. ISGylation was also found to be dysregulated in transformed hepatoma cell lines. Importantly, epigenetic regulation of SOCS1 and subsequently ISGylation may be important factors in the development of cell type-specific host defense responses in hepatocytes, which could also be implicated in the development of liver cancer.

## Materials and methods

See supplementary information for procedures on cell culture, hepatocyte differentiation, RNA sequencing (RNAseq), HCV infection, lipofectamine transfection, RNA interference, plasmid transfection, real-time quantitative PCR, immunoblot assay, immunoprecipitation assay, reduced representative bisulfate sequencing, and chromatin immunoprecipitation qPCR.

### Statistical methods

Mean and standard deviation values were calculated using Microsoft Excel. GraphPad Prism 9’s unpaired Student’s *t* test with Welch’s correction was used to determine *p* values. A *p* value <0.05 was considered significant. *∗p* <0.05, ∗*∗p <*0.001, ∗∗*∗p <*0.0001.

## Results

### ISGylation occurs in hepatocytes following HCV infection

**Fig. 1:**
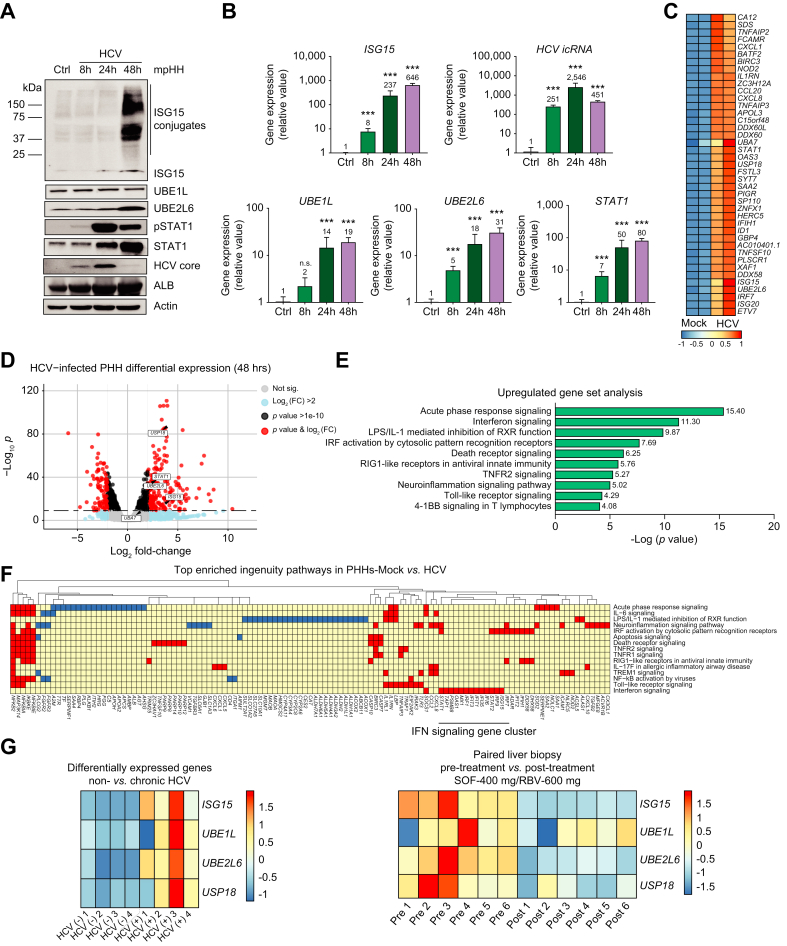
ISGylation is detectable in hepatocytes following HCV infection. (A-B) Mouse-propagated human hepatocytes (mpHHs) were treated with HCV (MOI=1) for 8, 24 and 48 h. (A) Western blot analysis of protein expression for ISG15, UBE1L, UBE2L6, pSTAT1, STAT1, HCV Core Protein and β-actin. (B) qPCR analysis of mRNA expression for *ISG15, UBE1L, UBE2L6, STAT1* and HCV intracellular RNA. Data from repeated experiments in triplicate were averaged and are expressed as mean and standard deviation values (error bar) presented with an unpaired Student’s *t* test with Welch’s correction used to determine the *p* values. A *p* value <0.05 was considered significant. *∗p <*0.05, ∗*∗p <*0.001, ∗∗*∗p <*0.0001, n.s., non-significant. (C-F) RNAseq analysis from PHHs following 48 h of HCV infection (MOI=1) https://www.ncbi.nlm.nih.gov/geo/query/acc.cgi?acc=GSE211161 (C) Heat map of gene expression from the top 40 significantly upregulated genes. (D) Volcano plot of gene expression changes stratified by log-fold change and *p* value. (E) Top 10 upregulated signaling pathways. (F) Top 15 enriched ingenuity pathways. (G-left panel) Heat map of gene expression of *ISG15, UBA7, UBE2L6* and *USP18* in individuals with and without chronic HCV infection from https://www.ncbi.nlm.nih.gov/geo/query/acc.cgi?acc=GSE84346 published dataset. (G-right panel) Heat map of gene expression of *ISG15, UBA7, UBE2L6* and *USP18* in humans pre- and post-treatment with sofosbuvir and ribavirin from https://www.ncbi.nlm.nih.gov/geo/query/acc.cgi?acc=GSE51699 published dataset. RNAseq data are from one experiment with two technical replicates. mpHHs, mouse-propagated human hepatocytes.

ISGylation is observed in multiple cell types in response to infection by many distinct pathogens; importantly, it has also been observed with the activation of cell-intrinsic innate antiviral responses, in primary human hepatocytes, following stimulation with a variety of pathogen-associated molecular patterns, HCV, and IFN treatment.^12^^,^^29^ For our initial studies, we sought to characterize the ISGylation system and corresponding expression of ISG15, UBE1L, UBE2L6, and STAT1 in mature hepatocytes following 48-hour stimulation with HCV. ISGylation was visualized through immunoblotting in which we observed a protein smear at varying molecular weights greater than the molecular weight of ISG15, which is approximately 15 kD. This smear represents the hundreds of mono-ISG15-conjugated proteins. ISGylation was observed at 48 h post HCV infection (Fig. 1A). Since ISGylation enzymes (and some targets) are also ISGs, we assessed the activation of STAT1, which is one of the main transcription factors that induces ISGs in response to type I and III IFN signaling.^4^^,^^6^ Increased ISGylation was observed following HCV infection with corresponding higher levels of ISG15, UBE2L6, STAT1 and activated STAT1 (pSTAT1) (Fig. 1A). Concurrently, we observed robust ISGylation at 48 h post infection with STAT1 activation (Fig. 1A). Increased expression of *ISG15, UBE1L, UBE2L6* and *STAT1* mRNA was also observed (Fig. 1B).

RNAseq analysis of PHHs 48 h after HCV infection also indicated that *ISG15, UBE1L (UBA7), UBE2L6,* and *STAT1* are among the top upregulated genes in an unbiased analysis (Fig. 1C–D). These components of the ISGylation system are primarily associated with the IFN signaling pathway (Fig. 1E and F). Utilizing RNAseq data from uninfected and chronically infected individuals,^30^ we observed that genes associated with ISGylation are upregulated during chronic HCV infection (Fig. 1G-left panel). In cured patients, we also observed the downregulation of genes associated with ISGylation following treatment with DAAs^31^ (Fig. 1G-right panel). Overall, this data demonstrates that components of the ISGylation system are upregulated during HCV infection.

### ISGylation is not detected in iPSCs following stimulation with viral mimetics and IFN

**Fig. 2:**
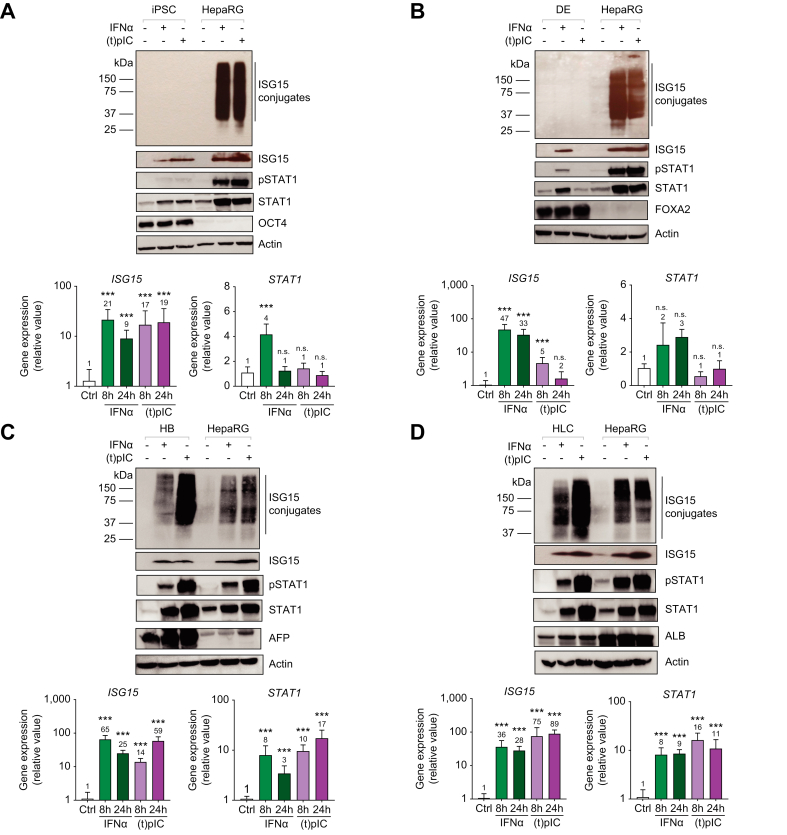
ISGylation is not detected in iPSCs following stimulation with IFNα or poly(I:C) but is detectable in HLCs. Cells were treated with 1,000 U/ml IFNα or 500 ng transfected poly(I:C)=(t)pIC for 8 (mRNA expression) and 24 h (mRNA and protein expression). (A-D, top panel) Western blot analysis for protein expression of ISG15, pSTAT1, STAT1, β-actin, and respective cell markers OCT4, FOXA2, AFP, and ALB in iPSCs, DEs, HBs, and HLCs, respectively. (A-D, bottom panel) qPCR analysis of mRNA expression for *ISG15* and *STAT1* in iPSCs, DEs, HBs and HLCs. Data from repeated experiments in triplicate were averaged and are expressed as mean and standard deviation values (error bar) presented with an unpaired Student’s *t* test with Welch’s correction used to determine the *p* values. A *p* value <0.05 was considered significant. *∗p <*0.05, ∗*∗p <*0.001, ∗∗*∗p <*0.0001, n.s., non-significant. (t)pIC, transfected poly(I:C).

Given the biologic significance of ISGylation during HCV infection, we next endeavored to understand the regulation of the ISGylation system during the differentiation of iPSCs to hepatocytes. Studies have demonstrated an attenuated IFN response in iPSCs ^27^^,^^28^; however, none have focused on the regulation of ISGylation in stem cells. Cells at each stage of hepatocyte differentiation (Fig. S1) including iPSCs (SLC101A), definitive endoderm, hepatoblasts (HBs), and hepatocyte-like cells (HLCs) were treated for 8 and 24 h with IFN or transfected poly(I:C). As a positive control, the HepaRG cell line was used since these cells reproducibly demonstrate a high level of ISGylation following stimulation. Analysis of stage-specific gene expression was also performed to confirm the differentiation process; OCT4 (octamer-binding transcription factor 4) was expressed at the iPSC stage, FOXA2 (forkhead box A2) was expressed at the definitive endoderm stage, AFP (alpha-fetoprotein) was expressed at the HB stage, and albumin was expressed at the HLC stage and in HepaRG cells (Fig. 2A-D-top panel). Baseline protein expression of ISG15 and subsequent upregulation following stimulation was observed at each stage of differentiation (Fig. 2A-D-top panel). This observation was further corroborated as *ISG15* mRNA levels significantly increased at 8 and 24-hours post stimulation (Fig 2A-D-bottom panel) at each stage. Interestingly, although the ISG15 monomer was detectable following stimulation at all the stages of differentiation, ISGylation was not observed in iPSCs after stimulation with IFN or transfected poly(I:C) (Fig. 2A-D-top panel). To further investigate the IFN signaling pathway at each stage, we characterized STAT1 and pSTAT1 protein expression. STAT1 protein was expressed and further upregulated following treatment with IFN or transfected poly(I:C) (Fig. 2A-D-top panel). This observation was supported as *STAT1* mRNA levels significantly increased (>3-fold) at 8 and 24-hours post IFN treatment (Fig. 2A-D-bottom panel). Although STAT1 expression was detected following IFN stimulation, very low to undetectable levels of activated STAT1 were observed in iPSCs when compared to HepaRG cells (Fig. 2A). This suggests that ISG15 is differentially regulated when compared to other ISGs in iPSCs. Studies have demonstrated that ISG15 expression is primarily regulated by the STAT2-IRF9 transcription factor complex independently of STAT1.^32^ This led us to further investigate the expression of the enzymes facilitating ISGylation, their relation to STAT1 and the expression of ISGylation substrates.

### Correlation between IFN-induced ISGylation, UBE2L6 expression, and STAT1 activation during hepatocyte differentiation

**Fig. 3:**
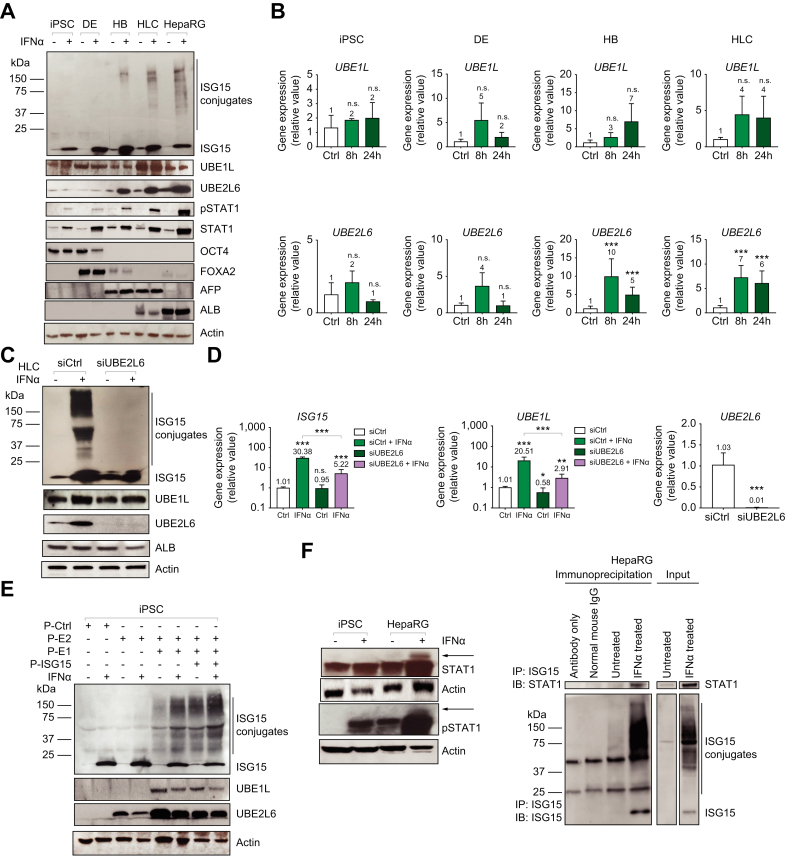
Correlation between IFN-induced ISGylation, UBE2L6 expression and STAT1 activation during iPSC differentiation to HLCs. (A) Cells were untreated or treated with 1,000 U/ml of IFNα for 24 h and western blot analysis was performed on iPSCs, DEs, HBs, HLCs and HepaRG cells for ISG15, UBE1L, UBE2L6, pSTAT1, STAT1, OCT4, FOXA2, AFP, ALB and β-actin. (B) qPCR analysis of mRNA expression for *UBE1L* and *UBE2L6*, at each stage of differentiation, where cells were treated with 1,000 U/ml of IFNα for 8 and 24 h. Data from repeated experiments in triplicate were averaged and are expressed as mean and standard deviation values (error bar) presented with an unpaired Student’s *t* test with Welch’s correction used to determine the *p* values. A *p* value <0.05 was considered significant. *∗p <*0.05, ∗*∗p <*0.001, ∗∗*∗p <*0.0001, n.s., non-significant. (C) Western blot analysis of UBE2L6 siRNA-treated HLCs where cells were treated with 50 μM UBE2L6 siRNA for 48 h before the addition of IFNα for an additional 24 h. (D) qPCR analysis of mRNA expression for *ISG15, UBE1L* and *UBE2L6* at 72 h post IFNα treatment. Data from repeated experiments in triplicate were averaged and are expressed as mean and standard deviation values (error bar) presented with an unpaired Student’s *t* test with Welch’s correction used to determine the *p* values. A *p* value <0.05 was considered significant. *∗p <*0.05, ∗*∗p <*0.001, ∗∗*∗p <*0.0001, n.s., non-significant. (E) Western blot analysis of overexpression using plasmids for ISG15, UBE2L6, and UBE1L. iPSCs were treated with 1 μg of transfected control, ISG15, UBE1L and UBE2L6 plasmid (P-=plasmid) for 24 h with IFNα treatment. (F) Western blot analysis of IFNα-treated iPSCs and HepaRG cells for STAT1 and pSTAT1 protein expression (left panel). IP assay in HepaRG cells (right panel). HepaRG cells were treated with IFNα followed by ISG15 IP and subsequent IB for ISG15 (bottom right panel) or STAT1 (top right panel) protein.

Given that, in iPSCs, ISG15 is upregulated following IFN treatment, we initially tested whether these cells were deficient in other components of the ISGylation system. Specifically, with a focus on IFN treatment, we directly compared protein expression levels of ISG15, the activating enzyme UBE1L and the conjugating enzyme UBE2L6, pSTAT1, STAT1, and stage markers in iPSC, DEs, HBs, and HLCs during differentiation. UBE1L protein expression was highest in HLCs (Fig 3A). Interestingly, there was no significant increase in *UBE1L* mRNA levels following IFN treatment at any stage of differentiation (Fig 3B-top panel). Analysis of UBE2L6 protein levels demonstrated weak expression in the iPSC and DEs with expression increasing in the latter stages (HB and HLC) of differentiation following IFN treatment (Fig. 3A). This was supported by *UBE2L6* mRNA levels increasing (>5-fold) following IFN treatment in HBs and HLCs (Fig. 3B- bottom panel). Likewise, there was no statistically significant increase in *UBE2L6* mRNA expression upon IFN treatment in iPSCs or DEs. Importantly, as UBE2L6 was minimally induced following IFN stimulation, pSTAT1 expression remained low, and ISGylation was not detected in iPSCs (Fig. 3A).

To determine if undetectable ISGylation in iPSCs is a consequence of the lack of induced UBE2L6 expression, we preformed targeted mechanistic studies utilizing plasmid overexpression and small-interfering RNA (siRNA) knockdown of UBE2L6 in iPSCs and HLCs, respectively. HLCs were treated with non-targeting control siRNA (siCtrl) or UBE2L6-targeted siRNA (siUBE2L6) for 48 h and subsequently treated with IFNα for an additional 24 h. As expected, our results demonstrated that siRNA knockdown of UBE2L6 decreased ISGylation to undetectable levels (Fig. 3C). qPCR revealed a 98.6% knockdown of *UBE2L6* mRNA expression at 72 h post siRNA treatment (Fig. 3D). Similar results were obtained for siRNA knockdown of ISG15 (Fig. S2A) In iPSCs, UBE2L6 was overexpressed through plasmid transfection (P-E2) either alone or in combination with a UBE1L expression plasmid (P-E1), or in combination with both UBE1L (P-E1) and ISG15 expression plasmids (P-ISG15) (Fig. 3E). Our results demonstrated that ISGylation occurred when UBE1L and UBE2L6 expression was increased, while the addition of ISG15 further increased ISGylation slightly in iPSCs, suggesting that the expression levels of UBE1L and UBE2L6 are not sufficient to facilitate the highest levels of ISGylation. This was confirmed in a different iPSC line, IMR90 (Fig. S2B).

We next assessed the expression of ISGylation target proteins to confirm that the lack of ISGylation in iPSCs was not due to an absence of the protein substrates of ISGylation. A well characterized protein that is covalently modified through attachment of 15 kDa ISG15 is the 88 kDa STAT1 protein. To demonstrate the ISGylation of STAT1, we observed the presence of a 15 kDa larger STAT1 protein band, through immunoblot analysis, in IFN-treated HepaRG cells that suggests that ISG15 is covalently conjugated to STAT1. Furthermore, we observed this larger band when immunoblotting for pSTAT1, suggesting that ISG15 is also conjugated to activated pSTAT1 following IFN treatment (Fig. 3F-left panel). To confirm the specific ISGylation of STAT1, ISG15 immunoprecipitation was performed on untreated and IFNα-treated HepaRG cells followed by immunoblotting for STAT1 and ISG15. Importantly, we were able to detect STAT1 in the concentrated pool of proteins covalently linked to ISG15 (Fig. 3F-right panel). In addition, when probed with an anti-ISG15 antibody, we found a broad smear confirming ISG15-conjugated proteins, which was further confirmed utilizing siRNA targeted to ISG15 in HLCs (Fig. S2A). Taken together, these results demonstrated that although iPSCs upregulated ISG15 following IFN treatment, these cells did not have detectable ISGylation. In contrast, HLCs demonstrated robust ISGylation, and STAT1 was ISGylated in HepaRG cells. Furthermore, we observed lower pSTAT1 expression in iPSCs suggesting that these cells have an attenuated response to IFN and this attenuation may underlie the lack of detectable ISGylation in iPSCs. Overall, our results revealed that the lack of ISGylation in iPSCs is correlated to the lack of IFN-induced expression of UBE2L6 and STAT1 activation. In addition, the expression of UBE1L, which is not regulated by IFN signaling in iPSCs, is not high enough at baseline to contribute to ISGylation.

### Baseline SOCS1 expression decreases as ISGylation increases during the differentiation of iPSCs to HLCs

To follow-up on the observation of low pSTAT1 levels following IFNα treatment in iPSCs, we next investigated inhibitors of IFN signaling. Initially, we tested kinase inhibitors in IFN-treated HepaRG cells (Fig. S2C–D). Utilizing a specific Janus kinase (JAK)1/2 inhibitor, we observed decreased ISGylation, ISG15, and STAT1 activation (Fig. S2C). However, when we treated cells with the broad tyrosine kinase inhibitor, ruxolitinib, we saw a complete downregulation of ISGylation, ISG15, STAT1 and pSTAT1 expression to baseline levels (Fig. S2D). This suggested that not only JAKs are involved in activating STAT1 and subsequently increasing ISG15 (and ISGylation), but that other tyrosine kinases are also involved in regulating STAT1 activation.

**Fig. 4:**
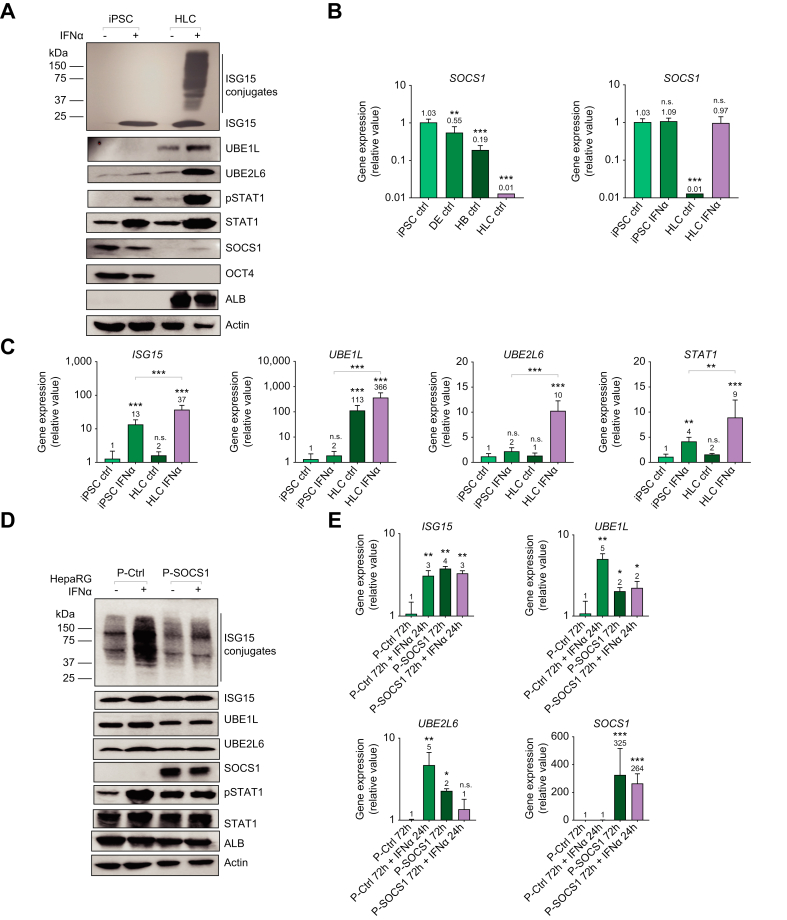
Baseline SOCS1 expression decreases as iPSCs differentiate to HLCs that display ISGylation. (A) Direct comparison of protein expression for ISG15, ISGylation, UBE1L, UBE2L6, pSTAT1, STAT1, SOCS1, OCT4, ALB and β-actin in iPSCs and HLCs. Cells were treated with 1,000 U/ml of IFNα for 24 h. (B) qPCR data for *SOCS1* mRNA basal level expression in iPSCs, DEs, HBs and HLCs (left panel) and in iPSCs and HLCs with and without 8 h of IFNα treatment (right panel). (C) qPCR data for mRNA expression of *ISG15, UBE1L, UBE2L6* and *STAT1* in iPSCs and HLCs 8 h post IFNα treatment. Data from repeated experiments in triplicate were averaged and are expressed as mean and standard deviation values (error bar) presented with an unpaired Student’s *t* test with Welch’s correction used to determine the *p* values. A *p* value <0.05 was considered significant. *∗p <*0.05, ∗*∗p <*0.001, ∗∗*∗p <*0.0001, n.s., non-significant. (D) Protein expression for ISG15, ISGylation, UBE1L, UBE2L6, SOCS1, pSTAT1, STAT1, β-actin and ALB following 72 h of transfection of 1 μg control or SOCS1 expression plasmid (P=plasmid) with 1,000 U/ml of IFNα for 24 h. (E) qPCR data for mRNA expression of *ISG15, SOCS1, UBE1L, UBE2L6* and *STAT1* with corresponding conditions as found in panel D. Data from repeated experiments in triplicate were averaged and are expressed as mean and standard deviation values (error bar) presented with an unpaired Student’s *t* test with Welch’s correction used to determine the *p* values. A *p* value <0.05 was considered significant. *∗p <*0.05, ∗*∗p <*0.001, ∗∗*∗p <*0.0001, n.s., non-significant.

As mentioned previously, Hong *et al.*^27^ demonstrated that iPSCs have an attenuated IFN response due to high intrinsic expression of the SOCS1 protein, which is an inhibitor of STAT1 phosphorylation attributed to blocking JAK1 and tyrosine kinase 2 activation. To determine the role of SOCS1 in regulating ISGylation, we directly compared iPSCs and HLCs for protein and mRNA expression of SOCS1 following 8 and 24 h of IFN stimulation. Constitutive SOCS1 mRNA and protein expression was detected both at baseline and upon IFN stimulation in iPSCs at 8 and 24 h. Baseline SOCS1 expression was not detected in HLCs and IFN treatment elicited only a minimal increase in SOCS1 protein expression compared to the levels seen in iPSCs (Fig. 4A). This suggests that SOCS1, in HLCs, is differentially regulated. During hepatocyte differentiation, we observed a gradual decrease in baseline *SOCS1* mRNA expression (Fig. 4B). UBE1L mRNA and protein expression was strikingly higher (>100-fold) in HLCs at both baseline and upon IFNα treatment (Fig. 4C). Overall, there is significantly higher mRNA expression of *ISG15, UBE1L, UBE2L6,* and *STAT1* following 8 h of IFN treatment in HLCs compared to iPSCs. Importantly, we observed a correlation between the baseline expression level of SOCS1, the degree of ISGylation, UBE2L6 expression, and STAT1 activation. Specifically, as baseline SOCS1 expression decreased, IFN-induced STAT1 activation, UBE2L6 expression, and ISGylation increased (Fig. 4 and Fig. S3B-C). This highlights the importance of baseline SOCS1 levels, prior to stimulation with IFN, suggesting that high baseline SOCS1 expression negatively regulates STAT1 activation and the subsequent increases in UBE2L6 expression needed to facilitate ISGylation. These results were further confirmed through a 48-hour IFNα time course experiment in iPSCs, HLCs, HepaRG cells, and mature hepatocytes (Fig. S4A-C). The protein and mRNA expression of the ISGylation-deconjugating enzyme USP18 was not correlated with ISGylation during hepatocyte differentiation. Specifically, *USP1*8 mRNA expression at baseline was similar between iPSCs and HLCs and significantly lower in DEs and HBs (Fig. S3A-B). This data suggests that USP18 and its ISGylation-deconjugating activity are not major factors in the inhibition of ISGylation in iPSCs and DEs.

To determine the effects of modulating SOCS1 expression on ISGylation levels, we first utilized HepaRG cells that were transfected with the SOCS1 expression vector (P-SOCS1) or an empty vector control (P-Ctrl). Forty-eight hours post transfection, the cells were stimulated with IFNα for 24 h. Although the level of ISGylation was increased upon IFN treatment in the control vector-treated cells, possibly through DNA sensing mechanisms,^29^ there was no apparent increase in ISGylation in the SOCS1-expressing cells after IFN treatment (Fig. 4D). Furthermore, increased SOCS1 expression prevented IFN-induced UBE2L6 protein expression and decreased mRNA expression two-fold when compared to control vector (Fig. 4D-E). IFN-induced UBE1L expression decreased by two-fold (Fig. 4E). These data revealed that the overexpression of SOCS1 resulted in an impairment in IFN-induced ISGylation by decreasing UBE2L6 and UBE1L levels.

### ISGylation observed with STAT1- dependent induced expression of UBE2L6 in iPSCs following SOCS1 siRNA knockdown

**Fig. 5:**
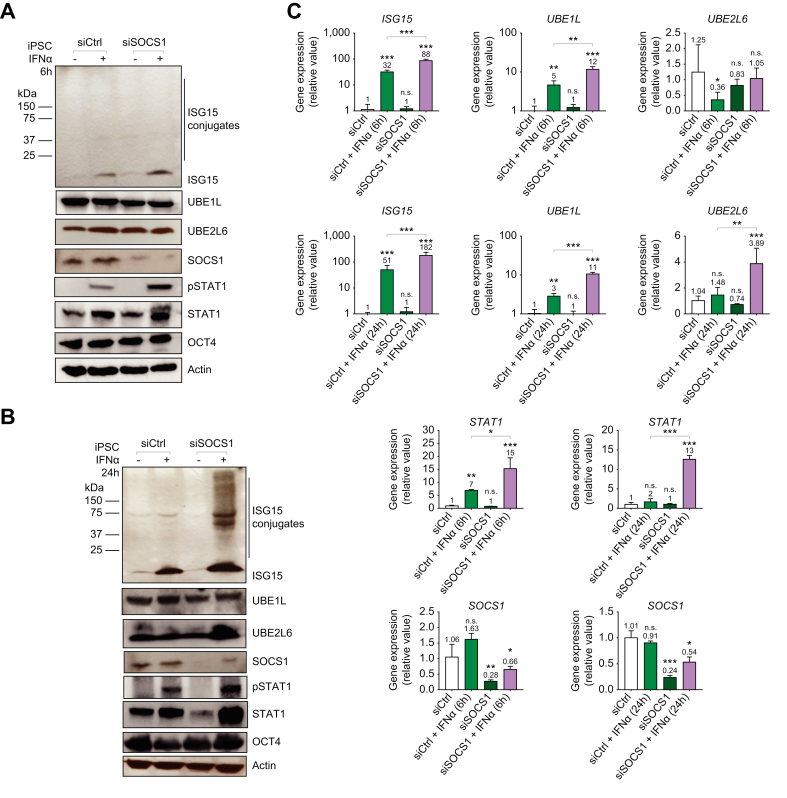
ISGylation observed with STAT1-dependent upregulation of UBE2L6 in iPSCs following SOCS1 siRNA knockdown. (A-C) iPSCs were transfected with 50 μM control or SOCS1 siRNA for 48 h (day 0-2), retreated at day two, and treated with 1,000 U/ml of IFNα on day four for 6 h or 24 h (day 5). (A-B) Protein expression for ISG15, ISGylation, UBE1L, UBE2L6, SOCS1, pSTAT1, STAT1, β-actin and OCT4. (C) qPCR analysis of mRNA expression for *ISG15, SOCS1, UBE1L, UBE2L6* and *STAT1* following 0, 6 or 24 h of IFNα stimulation. Data from repeated experiments in triplicate were averaged and are expressed as mean and standard deviation values (error bar) presented with an unpaired Student’s *t* test with Welch’s correction used to determine the *p* values. A *p* value <0.05 was considered significant. *∗p <*0.05, ∗*∗p <*0.001, ∗∗*∗p <*0.0001, n.s., non-significant.

To further assess the inhibitory effect of SOCS1 on ISGylation, we examined the impact of siRNA-mediated silencing of SOCS1 in iPSCs. iPSCs were treated with either non-targeting control siRNA (siCtrl) or siRNA targeting SOCS1 (siSOCS1) for 96 h (4 days) prior to treating cells with IFN. Samples were collected at either 6 or 24 h post IFNα treatment. At 6 h post IFNα treatment, ISGylation was not observed and there was no apparent difference in protein expression for UBE1L or UBE2L6 between siCtrl and siSOCS1 treatment conditions. However, higher ISG15, STAT1 and pSTAT1 protein expression levels were observed in the SOCS1-silenced cells following IFN treatment (Fig. 5A). At 24 h post IFN treatment, there was an elevation in ISG15, UBE2L6, pSTAT1, and STAT1 protein expression in the SOCS1-silenced cells treated with IFNα compared to the control siRNA-treated cells treated with IFNα (Fig. 5B). Importantly, ISGylation was enriched in the SOCS1-silenced cells treated with IFN. Furthermore, the mRNA expression for *ISG15, UBE1L,* and *STAT1* was significantly higher in the IFN-treated SOCS1 condition when compared to the IFN-treated siCtrl control condition at both the 6 h and 24 h time points post IFNα treatment (Fig 5C). Importantly, *UBE2L6* mRNA expression was significantly higher (4-fold) in the IFN-treated SOCS1 condition when compared to the IFN-treated siCtrl control condition at 24 h. The efficiency of SOCS1 knockdown was determined to be approximately 74% (Fig. 5C). These data demonstrated that downregulating SOCS1 expression resulted in the restoration of STAT1-dependent ISGylation. This was through the increased expression of UBE2L6 and pSTAT1 in siSOCS1 treated cells. There is a delay in UBE2L6 upregulation and STAT1 activation, in SOCS1 expressing iPSCs, that persisted over the 24 h of IFN treatment. This delayed increase in UBE2L6 expression following STAT1 activation was demonstrated in our IFN time course experiment (Fig. S4). With greater SOCS1 knockdown, we would expect a greater increase in IFN-induced expression in UBE2L6 and increased ISGylation.

### High SOCS1 expression in iPSCs attributed to epigenetic regulation

**Fig. 6:**
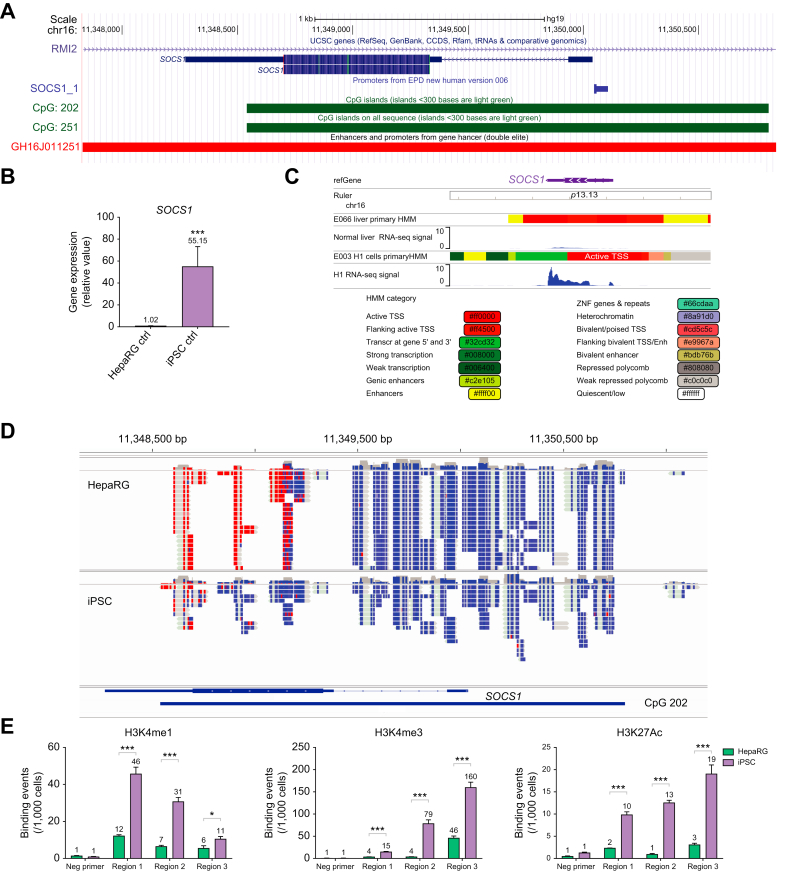
Increased SOCS1 expression in iPSCs attributed to epigenetic regulation. (A) UCSC Genome Browser screenshot of *SOCS1* gene with promoter, CpG island and Genehancer tracks displayed (B) qPCR analysis of mRNA levels for baseline expression of SOCS1 in iPSCs and HepaRG cells. Data from repeated experiments in triplicate were averaged and are expressed as mean and standard deviation values (error bar) presented with an unpaired Student’s *t* test with Welch’s correction used to determine the *p* values. A *p* value <0.05 was considered significant. *∗p <*0.05, ∗*∗p <*0.001, ∗∗*∗p <*0.0001, n.s., non-significant. (C) ENCODE data on RNA sequencing for *SOCS1* gene expression in H1 embryonic stem cells and in liver cells with legend for colored-coded gene regulatory elements. (D) Reduced representation bisulfate sequencing for DNA methylation status of *SOCS1* gene (mapped to GRCh37/hg19) in iPSCs and HepaRG cells. Red indicates methylated CpG bases and blue indicates unmethylated CpG bases. (E) qPCR analysis of chromatin-immunoprecipitation for binding events of histone modifications H3K4me1, H3K4me3, and H3K27ac in iPSCs and HepaRG cells. Three genomic regions of *SOCS1* gene were mapped to GRCh37/hg19; Region 1: 11,347,874-11,348,373bp. Region 2: 11,348,390-11,348,889bp. Region 3: 11,348,920-11,349,444bp in comparison to data using a negative (Neg) control primer. Data from repeated experiments in triplicate were averaged and are expressed as mean and standard deviation values (error bar) presented with an unpaired Student’s *t* test with Welch’s correction used to determine the *p* values. A *p* value <0.05 was considered significant. *∗p <*0.05, ∗*∗p <*0.001, ∗∗*∗p <*0.0001, n.s., non-significant. ENCODE integrative analysis (PMID: 22955616; PMCID: PMC3439153) ENCODE portal (PMID: 29126249; PMCID: PMC5753278). UCSC Genome Browser SessionURL(SOCS1):http://genome.ucsc.edu/cgi-bin/hgTracks?db=hg19&lastVirtModeType=default&lastVirtModeExtraState=&virtModeType=default&virtMode=0&nonVirtPosition=&position=chr16%3A11347831%2D11350479&hgsid=1280379467_j7ViSdKoDWUyqakJN2IuPyL2H5Yz.

To study the mechanisms underlying the dynamic regulation of SOCS1 during differentiation, we utilized online genome databases including the University of California Santa Cruz (UCSC) Genome Browser^33^ and data from the ENCODE project.^34^ Using the UCSC Genome Browser, we identified the genomic region of the *SOCS1* gene, its promoter and enhancer regions, and CpG island (CpG202) (Fig. 6A). Using our own samples from iPSCs and HepaRG cells we confirmed that SOCS1 levels were elevated (55-fold) in stem cells compared to hepatocytes (Fig. 6B). ENCODE data from H1 embryonic stem cell (ESC) lines and normal human liver cells confirmed the higher mRNA expression and genomic structural data were analyzed to identify the transcriptional regulatory structure of the *SOCS1* gene. This analysis revealed the presence of active promoter regions (pink, brown) surrounding the transcription start site (red) in the H1 ESCs that were not found in the primary liver samples. In addition, active enhancer elements (yellow) and transcription reported regions (light and dark green) were found in the H1 ESCs towards the 3’ end of the *SOCS1* gene that were not found in primary liver samples (Fig. 6C).

Reduced representation bisulfate sequencing revealed very little difference in DNA methylation status at the *SOCS1* promoter region in iPSCs and HepaRG cells (Fig. 6D). However, there were notable differences in the methylation status in the enhancer regions found at the 3’ end of the *SOCS1* gene in iPSCs compared to HepaRG cells (Fig. 6D). Specifically, the 3’ enhancer region, associated with the CpG island 202 and exon 2 of the *SOCS1* gene, contained primarily unmethylated DNA in iPSCs (associated with active transcription, blue color). This same region contained methylated DNA in HepaRG cells (associated with transcriptional repression, red color). Data from SOCS1-targeted chromatin co-immunoprecipitation qPCR revealed that the histone modifications H3K4me1, H3K4me3, and H3K27ac (associated with active promoters and/or enhancers) were enriched (>6-fold) in the *SOCS1* gene enhancer region in iPSCs compared to HepaRG cells (Fig. 6E). Furthermore, these significant differences in histone modifications and DNA methylation occurred within the same genomic region of the *SOCS1* gene (11,348,500-11,349,500 base pair range). Taken together, analysis of the transcriptional regulatory structure, DNA methylation status, and histone modifications of SOCS1 implicate epigenetic regulation in the downregulation of SOCS1 expression during hepatocyte differentiation.

Due to the observation of a significant gradual increase in baseline UBE1L expression as stem cells differentiated to hepatocytes (Fig. S5A-B), we also compared *UBE1L* gene regulatory regions in H1 ESCs to primary hepatocytes using data from ENCODE. In this case, the transcribed region (green) and transcription start site region (red-orange) were observed at the 5’ end of the *UBA7 (UBE1L*) gene in primary hepatocytes and not ESCs, suggesting epigenetic regulation is occurring to the 5’ promoter and/or gene body region of *UBA7* (Fig. S5C). Utilizing the CpG Methylation by Methyl 450K Bead Arrays track on the UCSC genome browser, we observed methylation marks (orange) in ESCs and unmethylated marks in hepatocytes (blue) at the promoter region and within exon 24, further suggesting epigenetics also regulates baseline expression of *UBE1L* during hepatocyte differentiation (Fig. S5D).

Overall, based on these series of experiments and *in silico* data for SOCS1, high baseline SOCS1 expression in iPSCs inhibits IFN-induced ISGylation by blocking STAT1 activation and subsequent upregulation of ISGs like UBE2L6. Furthermore, low baseline SOCS1 expression in hepatocytes facilitates IFN-induced STAT1-dependent ISGylation. ISG15 expression is regulated by STAT1-independent IFN signaling, UBE2L6 expression is regulated by STAT1-dependent IFN signaling (that is negatively regulated by SOCS1) and UBE1L expression is regulated through epigenetic mechanisms.

### High SOCS1 expression is observed in the HepG2- and Huh 7.5.1-transformed hepatoma cell lines

**Fig. 7:**
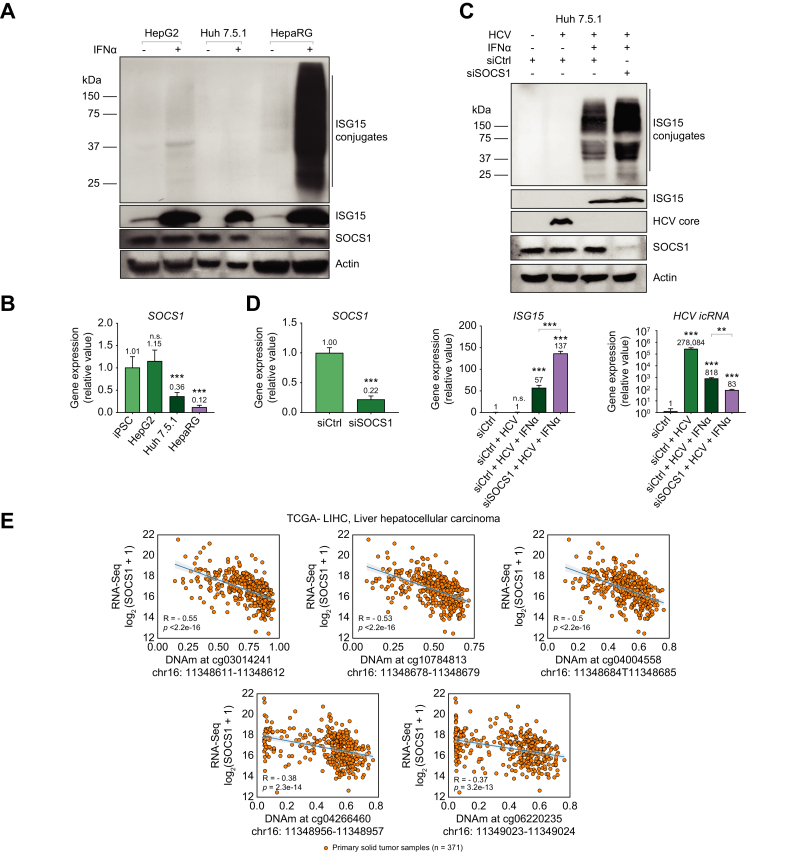
Increased baseline SOCS1 expression observed in the transformed HepG2 and Huh 7.5.1 hepatoma cell lines. (A) Western blot analysis comparing protein expression for ISG15, ISGylation, SOCS1, and β-actin in HepG2, Huh 7.5.1 and HepaRG cells. Cells were treated with 1,000 U/ml of IFNα or 500 ng of transfected poly(I:C)=(t)pIC for 24 h. (B) qPCR analysis of mRNA expression for *SOCS1* at baseline in HepG2, Huh 7.5.1, and HepaRG cells compared to iPSCs. Data from repeated experiments in triplicate were averaged and are expressed as mean and standard deviation values (error bar) presented with an unpaired Student’s *t* test with Welch’s correction used to determine the *p* values. A *p* value <0.05 was considered significant. *∗p <*0.05, ∗*∗p <*0.001, ∗∗*∗p <*0.0001, n.s., non-significant. (C) Western blot analysis for ISG15, SOCS1 and HCV Core protein expression. (D) qPCR analysis of mRNA expression for SOCS1 siRNA knockdown efficiency, mRNA expression for ISG15 and intracellular HCV viral RNA. (C-D) Cells were treated with 50 μM of non-targeting siRNA control or siRNA targeting SOCS1 for two days prior to retreating cells with 50 μM siRNA control or SOCS1 in addition to HCV infection (MOI = 1) alone, or HCV and 1,000 U/ml of IFNα concomitantly for 48 h. Data from repeated experiments in triplicate were averaged and are expressed as mean and standard deviation values (error bar) presented with an unpaired Student’s *t* test with Welch’s correction used to determine the *p* values. A *p* value <0.05 was considered significant. *∗p <*0.05, ∗*∗p <*0.001, ∗∗*∗p <*0.0001, n.s., non-significant. (E) Graphs plotting DNA methylation percentages at the 3’ genomic region of the *SOCS1* gene *vs*. mRNA expression of *SOCS1* based on RNA sequencing in Log_2_ scale. R value and *p* value determined by best fit line from 371 primary HCC solid tumor samples. (t)pIC, transfected poly(I:C).

In mature human hepatocytes, we demonstrated a correlation between increased ISGylation and decreased HCV replication (Fig. 1A) at a very early time point. Although these cells are an optimal model for *in vitro* modeling of HCV infection, immortalized hepatoma cell lines have been frequently utilized to study HCV infection. In addition, they have been used to propagate virus as these transformed cells are highly permissive to HCV infection in part due to dysregulated antiviral responses. Intriguingly, when we investigated ISGylation in the Huh 7.5.1 and HepG2 cell lines, we observed minimal ISGylation following IFN stimulation (Fig. 7A). When we evaluated SOCS1 protein expression in these transformed cell lines, we detected higher baseline SOCS1 protein expression when compared to HepaRG cells that are not transformed but only immortalized. When compared to iPSCs, baseline mRNA expression revealed no significant difference when compared to levels seen in HepG2 cells (Fig. 7B). Similar to iPSCs, ISGylation was not detected after IFN treatment in HepG2 cells. *SOCS1* mRNA expression in Huh 7.5.1 was lower than in iPSCs and HepG2 cells but higher than in HepaRG cells (Fig. 7B). This coincided with observably lower ISGylation in response to IFN treatment in Huh 7.5.1 cells compared to HepaRG cells. This data further supports our finding that SOCS1 represses ISGylation.

Since ISGylation occurs following HCV infection of mature hepatocytes, we investigated the effects of modulating SOCS1 expression on ISGylation, using siRNA during HCV infection, in Huh 7.5.1 cells. Huh 7.5.1 cells have a defect in RIG-I associated viral RNA sensing and subsequent IRF3 activation ^35^; therefore, HCV infection does not stimulate IFN signaling and ISGylation was not observed following 48 h HCV infection alone. Concurrently, HCV core protein was highly expressed (Fig. 7C). With the addition of IFNα treatment, ISGylation was observed, and HCV core protein was not detected at this early time point. The addition of siRNA targeting SOCS1 with IFN treatment and HCV infection increased ISGylation compared to the siRNA control condition. Low SOCS1 protein expression was detected with siRNA knockdown. We achieved a 78% knockdown efficiency of *SOCS1* mRNA (Fig. 7D). Compared to the control siRNA condition, we observed a significant increase (2.5-fold difference) in *ISG1*5 mRNA following SOCS1 knockdown (Fig. 7D). Despite the downregulation of HCV intracellular RNA (HCV icRNA) (340-fold decrease) following IFN treatment without decreased SOCS1 expression, we observed a greater significant decrease (additional 3.5-fold decrease and 3,350-fold difference from HCV only condition) in HCV icRNA with *SOCS1* mRNA knockdown. With greater *SOCS1* mRNA knockdown efficiency, we would expect a further increase in ISGylation and greater downregulation of HCV icRNA. Taken together, this data demonstrated that decreased SOCS1 expression increases the antiviral effect of IFN and increases levels of ISGylation.

Lastly, the silencing of SOCS1 expression observed in hepatocytes compared to stem cells (Fig. 6) has also been observed through changes in DNA methylation in tumor cells. Therefore, we utilized The Cancer Genome Atlas database to obtain relevant data on *SOCS1* mRNA expression and DNA methylation patterns in the similar SOCS1 genomic regions, studied in Fig. 6D, in patient-derived liver tumor samples.^36^ When we analyzed RNA data from primary human HCC tumors, we observed varying levels of *SOCS1* mRNA expression (Fig. 7E). Importantly, this data supported the previously mentioned hypothesis that SOCS1 expression levels are inversely correlated with DNA methylation levels in the *SOCS1* gene 3’ enhancer region. Importantly, we were able to observe a moderate correlation (R = -0.37 to -0.55) between high DNA methylation and decreased *SOCS1* mRNA expression in HCC. Considering the *p* values (*p* <10^-13^ to 10^-16^) obtained from this data, this negative correlation is statistically significant.

## Discussion

In this manuscript, we explored the regulation of ISGylation, which is an important component of host defense responses to viral infection.^17^^,^^37^ We demonstrated that ISGylation is a primary component of the hepatocyte response to HCV infection (Fig. 1). Furthermore, we revealed a correlation between increased ISGylation and decreased HCV core protein and viral RNA levels. This observation can possibly be attributed to ISGylation of HCV viral protein NS5A as previously reported.^23^ Through characterization of protein and mRNA expression for ISG15, UBE1L, UBE2L6, STAT1, and pSTAT1 during iPSC differentiation to hepatocytes, we determined that ISGylation was undetectable in iPSCs (Fig. 2, Fig. 3). Furthermore, we demonstrated a lack of IFN-induced UBE2L6 expression and low levels of IFN-stimulated pSTAT1 in iPSCs. In addition, baseline expression of UBE1L was significantly lower in iPSCs compared to HLCs (Fig. 4). We demonstrated that targeted siRNA knockdown of *UBE2L6* mRNA inhibited ISGylation in HepaRG cells and HLCs while overexpression of UBE1L and UBE2L6 facilitated ISGylation in iPSCs (Fig. 3 and Fig. S2). Through targeted mechanistic studies, we demonstrated that high baseline expression of SOCS1 negatively regulates STAT1 activation and subsequently expression of UBE2L6 (Fig. 4, Fig. 5, Fig. S3-4, S6). Specifically, overexpression of SOCS1 diminished ISGylation, UBE2L6, and pSTAT1 levels in HepaRG cells (Fig. 4) while *SOCS1* mRNA knockdown facilitated STAT1 activation which subsequently led to detectable ISGylation in iPSCs (Fig. 5). We successfully differentiated iPSCs to HLCs and obtained similar results when characterizing the IFN response and ISGylation during hepatocyte differentiation in a different iPSC cell line, IMR90 (Fig. S6).

Given the dramatic difference in *SOCS1* and *UBE1L* baseline mRNA expression between iPSCs and HLCs, we explored mechanisms governing *SOCS1* transcription. The *SOCS1* gene in HepaRG cells was methylated at CpG sites within the 3’ gene body region which is indicative of repressed transcription (Fig 6). The *UBE1L (UBA7*) gene in iPSCs was methylated at the promoter region and within exon 24 of the gene body which is indicative of repressed transcription (Fig. S5). Studies have previously demonstrated that epigenetic regulation of an intragenic region (or “gene body”) containing a CpG island can vary based on cell type. Specifically, a gene may contain a methylated CpG island in one cell type and an unmethylated CpG island in another cell type. It is important to note that genes containing a CpG Island within their gene body, like SOCS1, have been implicated in differential regulation during differentiation.^38^ Based on our data, during hepatocyte differentiation, the differences in DNA methylation status of the intragenic CpG sites or CpG sites within the promoter region contribute to the differential expression of *SOCS1* and *UBE1L,* respectively. In addition, there are other pre- and post-transcriptional factors that can affect baseline mRNA expression such as microRNAs that inhibit expression^39^ and transcription factors that promote expression.^40^ Overall, this study demonstrates, for the first time, that SOCS1 negatively regulates ISGylation in a STAT1-UBE2L6-dependent manner. In stem cells, baseline *SOCS1* mRNA expression levels are increased (Fig. 4B) due, in large part, to epigenetic regulation (Fig. 6).

Interestingly, changes in SOCS1 expression through altered DNA methylation levels in tumor cells are well-characterized; however, complex phenotypes are observed with these changes. In the case of the cirrhotic liver, downregulation of SOCS1 has been associated with dysregulation of IFN signaling leading to an uncontrolled inflammatory response and to subsequent increased levels of fibrosis. Yoshida *et al.*^41^ demonstrated the correlation between low SOCS1 expression and *SOCS1* DNA hypermethylation in individuals at later stages of HCV-related cirrhosis, before HCC onset, suggesting that decreased SOCS1 expression may foster a pre-malignant state. In the case of HCC, the complexity is further illustrated by evidence implicating SOCS1 as both a tumor suppressor and oncogene based on its level of expression in tumor cells. These findings demonstrate that higher expression, as seen in stem cells, is related to oncogenic function in tumor growth and metastasis through its negative regulation of cell-autonomous IFNs, which have anti-cancer (anti-proliferative and pro-apoptotic) effects in tumor cells.^42^ Additional studies on SOCS1 expression and the ISGylation system, in HCC, may clarify the mechanisms underpinning their roles in the development of liver cancer, which may differ between the pre-malignant and malignant states.

Our studies investigating the lack of observable or decreased ISGylation in our transformed cell lines (Fig. 7A) revealed high SOCS1 expression in this setting. HCV replicates efficiently in these cell lines and does not on its own induce robust ISGylation (Fig. 7B).^43^ In Huh 7.5.1 cells, we demonstrated that treating these infected cells with IFN induces ISGylation that occurs concomitantly with a decrease in HCV replication (Fig. 7). This is interesting as neither treatment alone (IFN or HCV) induce robust ISGylation in Huh7.5.1 cells. However, ISGylation can be observed with concomitant stimulation with both HCV and IFN. Furthermore, siRNA-mediated SOCS1 knockdown further increased ISGylation levels. Additional studies are needed to further clarify the regulation of ISGylation and SOCS1 during both acute and chronic HCV infection in individuals with varying genetic backgrounds to account for polymorphisms at the type III IFN locus.^44^ Moreover, higher baseline SOCS1 expression may be an indicator of malignant transformation, in some tumors, arising from a more “stem cell-like” transcriptional program possibly attributed to epigenetic modifications.^45^

Intriguingly iPSCs, that have infinite replication potential, have high levels of SOCS1. This study and others have demonstrated that stem cells characteristically have attenuated IFN responses due to high expression of SOCS1. We demonstrated that SOCS1 is a strong negatively regulator to ISGylation. It is possible that additional negative regulators of the IFN pathway, that are specifically upregulated in stem cells, may also contribute to the lack of observed ISGylation.

In conclusion, ISGylation plays many roles in cell maintenance, function, and immunity.^19^^,^^22^^,^^37^ In the liver, ISGylation also has functions in metabolism.^18^ Like IFN signaling, ISGylation is involved in anti-proliferative activities and apoptotic cell death during host defense responses attributed to the stabilization of the tumor suppressor gene p53.[46], [47], [48] The absence of ISGylation, arising from increased SOCS1 expression in stem cells, can be a mechanism to maintain their self-renewal during development. Wu *et al.*^28^ reported that stem cells have a specific set of highly expressed ISGs that facilitate intrinsic antiviral functions instead of relying on delayed IFN-induced responses, as seen in terminally differentiated epithelial cells including hepatocytes.^2^^,^^6^^,^^14^ Furthermore, the liver undergoes dynamic changes during development. The fetal liver functions as an immune organ by housing hematopoietic stem cells before this reservoir migrates to the bone marrow.^49^ Studies have demonstrated that SOCS1 functions to retain hematopoietic stem cells in their undifferentiated state while IFN counteracts this activity by promoting differentiation.^50^ Overall, this study demonstrated that the development of cell-intrinsic innate immunity during the differentiation of iPSCs to hepatocytes provides insight into cell type-specific regulation of host defense responses and related oncogenic processes.

## Financial support

This works was supported by 10.13039/100000002NIH-10.13039/100000057NIGMS grants and fellowships; R35GM124915 principal investigator grant, R35GM124915S1 diversity supplement, R25GM076419 Initiative for Maximizing Student Diversity fellowship.

## Authors’ contributions

Concept & Design: Jasmine Edwards, Emmanuel Thomas. Acquisition of data: Jasmine Edwards, Stephanie Delabat, Robert DiCaprio, Robert Burgess, Jinhee Hyun, Steven Chen, Lily Wang, Emmanuel Thomas. Analysis and Interpretation: Jasmine Edwards, Stephanie Delabat, Robert DiCaprio, Alejandro Badilla, Robert Burgess, Jinhee Hyun, Stephen Chen, Lily Wang, Emmanuel Thomas. Manuscript Draft: Jasmine Edwards, Emmanuel Thomas. Manuscript Review: Jasmine Edwards, Alejandro Badilla, Derek Dykxhoorn, Emmanuel Thomas. Statistical Analysis: Jasmine Edwards, Stephanie Delabat, Alejandro Badilla. Funding acquisition: Jasmine Edwards, Emmanuel Thomas. Support: Derek M. Dykxhoorn, Takeshi Saito, Emmanuel Thomas Active Motif. Supervision: Emmanuel Thomas.

## Data availability statement

The RNA sequencing data is available to the public using the specific Gene Expression Omnibus accession number.

## Conflict of interest

The authors declare no conflict of interest.

Please refer to the accompanying ICMJE disclosure forms for further details.
