# Supplementary material for: Downregulation of SOCS1 increases interferon-induced ISGylation during differentiation of induced-pluripotent stem cells to hepatocytes
Source: JHEP Rep. 2022 Sep 23;4(12):100592. doi: 10.1016/j.jhepr.2022.100592 (PMC9685392; doi:10.1016/j.jhepr.2022.100592)
Supplement: Multimedia component 1 [file mmc1.pdf]

Downregulation of SOCS1 increases interferon-induced ISGylation during differentiation of induced-pluripotent stem cells to hepatocytes

Jasmine S Edwards, Stephanie A Delabat, Alejandro D Badilla, Robert C DiCaprio, Jinhee Hyun, Robert A Burgess, Tiago Silva, Derek M Dykxhoorn, Stephen Xi Chen, Lily Wang, Yuji Ishida, Takeshi Saito, Emmanuel Thomas

Table of contents

Supplementary methods..2

Supplementary figures...10

Supplementary references...22

I. Supplementary Methods

Cell Culture and Reagents

HepaRG cells (ThermoFisher Scientific) were maintained in William's E media supplemented with 10% HepaRG Growth Supplement (Lonza, ADD711C), 1% Glutamax, and 1% Pen/Strep. Huh 7.5.1 were provided by F. Chisari (Scripps Research Institute, La Jolla, CA) and HepG2 cells (ATCC, HB-8065) were maintained in DMEM media supplemented with 10% FBS, 1% Glutamax, and 1% antibiotics. Mouse passaged human hepatocytes (mpHHs) were were freshly isolated from chimeric mice that have humanized liver reconstituted with PHHs. The recovered mpHH were cultured in DMEM supplemented with 10% heat-inactivated FBS, 15μg/ml L-proline, 25ng/ml insulin, 50nM Dexamethasone, 5ng/ml, EGF, and 0.1mM L-ascorbic acid 2-phospate, 2% (v/v) DMSO as described previously [1]. PHHs were obtained from David Geller, at the University of Pittsburgh, through the NIH funded Liver Tissue and Cell Distribution System (LTCDS) and maintained in dHCGM media. Human iPSCs were maintained in 6-well tissue culture plates coated with Growth-Factor Reduced Matrigel. Cells were feed with mTESR$^+$ media supplemented with provided 5x mTESR$^+$ supplement and supplemented with 1% Antibiotic-Antimycotic solution. When passaging, 10mM Y27 and 10mM THZ molecules were added at 10μM and 1μM respectively to the feeding media then removed when media was replaced the next day**.** All cells were maintained at 37°C in humidified incubators with 5% CO_2.

iPSC Differentiation to Hepatocytes

Definitive Endoderm cells were maintained in RPMI 1640 Media supplemented with 10% B27 supplement, 1% Pen/Strep, and 1% Glutamax for 3 days with decreasing concentrations of the small molecule CHIR99021 (10μM, 3μM, 0μM). Hepatoblasts were maintained in DMEM Knockout Serum Replacement media supplemented with 1x MEM NEAA, 20% KO serum replacement serum, 5mM (2.5%)

Glutamax, and 1% Pen/Strep, 1% DMSO, and 0.001% [14.3M] β-Mercaptoethanol for 6 days. Hepatocyte-like cells were maintained in Leibovitz's L-15 Medium supplemented with 10% Tryptose phosphate broth, 10% FBS, 0.58% insulin-transferrin-selenium solution, 2mM (1%) Glutamax, 1% Pen/Strep, and small molecules sodium-L-ascorbate (50µg/mL), hydrocortisone-21-hemisuccinate (10µM), Dihexa (100nM), and Dexamethasone (100nM) for at least 8 days. All small molecules (expect sodium-L-ascorbate- diluted in PBS) were diluted in DMSO **(Fig. S1)**. All cells maintained at 37°C in humidified incubators with 5% CO_2. Stem cell lines SC101A and IMR90 were kindly provided by Dr. Dykxhoorn (University of Miami) [2].

HCV Infection

HCV JFH-1 was obtained from Dr. Jake Liang (NIH) and propagated as previously described [3] in Huh 7.5.1 cells. Primary Human Hepatocytes or Huh 7.5.1 cells were infected with HCV at an MOI of 1, unless otherwise indicated.

RNA Sequencing (RNAseq)

Total RNA from cells were isolated using the RNeasy Mini Kit with DNase treatment (Qiagen, 74106). RNA was quantified using Qubit (Thermo Fischer Scientific) and RNA integrity (RIN) was analyzed using BioAnalyzer 2100 lab-on-chip (Agilent) for a RIN score >7.0. Stranded total-RNA RNA-Seq libraries were synthesized using Illumina TruSeq Total Stranded with RiboZero Gold ribosomal RNA depletion. Resulting libraries were quantified and balanced using Qubit (Thermo Fischer Scientific), BioAnalyzer HS lab-on-chip (Agilent), and qPCR (Roche/Kapa). Paired end 2x 75 bp sequencing reads were carried out on the Illumina NextSeq 500 with samples pooled to generate a minimum of 40M PE75 reads per sample. Sequencing performance and raw data quality was monitored using Illumina SAV. After image analysis,

the raw read files (in fastq format) were analyzed using bioinformatics computational pipeline. Alignment, splice junction, and novel transcript identifications were performed using STAR aligner, with *UCSC human (mouse) genome ver. hg38 (mm10) and GENCODE gene annotation ver. GRCh38.p7 (GRCm38.p5) as references*. Feature Counts was used for counting number of reads per transcript. Post-alignment quality assessment was performed using Picard tools (http://broadinstitute.github.io/picard). RStudio (v2021.09.1 build 372) software utilizing R environment v4.1.2 is used for post-DESeq2 analysis in plotting DEGs. Heatmaps for top significant upregulated DEGs are plotted using pHeatmaps package (Pretty Heatmaps. R package version 1.0. 12. *R Package. version 1.0*, *8*.) after sorting upregulated genes by three parameters: adjusted p-value < 0.01, fold change >2, and Wald Chi-squared Test. Gene-wise p-values were computed using the *DESeq2* or *limma* package. Upregulated genes were ranked according to Wald statistic comparing Mock and HCV treated conditions of PHHs. Z-scores were calculated for gene counts of replicate conditions. The top 40 genes - including target genes ISG15, UBE2L6, UBA7 (UBE1L), and STAT1- were then plotted using pHeatmap package. Complete gene counts were then plotted using Enhanced Volcano package (https://github.com/kevinblighe/EnhancedVolcano) by the $-Log_{10}$(p-value) against Log_2 fold Change. Significance parameters were set in the volcano plot as $p < 1x10^{-10}$ and Log_{2f} fold change > 2. Standard pathway analysis based on differential expression results was performed using Ingenuity Pathway Analysis (IPA, QIAGEN Redwood City) and/or GSEA.

Viral Mimetic Lipofectamine Transfection and IFN treatment

Cells were seeded in 12-well tissue culture plates at a concentration of 1,000,000 cells/well for HLCs and 500,000 cells/well for iPSCs and HepaRG cells. Cells were treated at 80-90% confluency for indicated times. Lipofectamine 2000™ (ThermoFischer Scientific) was used to transfect 500ng of high molecular weight polyinosinic-polycytidylic acid [poly(I:C), Invivogen, tlrl-pic] into the target cells. IFNα

(Human Interferon- α2b, PBL Assay Science, 11105-1) was added directly to culture media at a concentration of 1000U/mL.

RNA Interference

Cells were seeded in 12-well tissue culture plates at a concentration of 800,000 cells/well for HLCs and 250,000 cells/well for iPSCs and HepaRG cells. Cells were treated at 70-80% confluency. siRNAs (50µM) were transfected into the appropriate cells using RNAiMax (Thermo Fisher Scientific) following the manufacturer's protocol. IFNα was added directly to culture media for indicated time prior to sample collection. All human siRNAs – SOCS1 (M-011511-04-0005), ISG15 (M-004235-04-0005), UBE2L6 (M-008569-02-0005), and non-targeting control (D-001206-13-20) – were purchased as siGENOME siRNA SMARTpool were purchased from Horizon Discovery and prepared according to manufacturer's instructions.

Plasmid Transfection

Cells were seeded in 12-well tissue culture plates at a concentration of 800,000 cells/well for HLCs and 450,000 cells/well for iPSCs and HepaRG cells. Cells were treated at 60-80% confluency at the appropriate time as indicated. Cells were treated with 1µg of plasmid using Lipofectamine™ 2000 or Lipofeactamine™ Stem Transfection Reagent (ThermoFisher Scientific). IFNα was added directly to culture media for the indicated time prior to sample collection. Plasmids were purchased from Origene and prepared according to manufacturer's instructions: ISG15 (NM_005101) Human Tagged ORF Clone, Ube1L (UBA7) (NM_003335) Human Tagged ORF Clone, UBE2L6 (NM_004223) Human Tagged ORF Clone, SOCS1 (NM_003745) Human Tagged ORF Clone, pCMV6-Entry (PS100001) Mammalian Expression Vector.

Real Time Quantitative PCR

TaqMan Probes for quantitative Real Time PCR (qRT-PCR) analysis were purchased from Integrated DNA Technologies (ISG15, UBE1L, UBE2L6, STAT1, SOCS1, OCT4, FOXA2, AFP, and albumin). Total RNA from cells were isolated using TRIzol according to the manufacturer's protocol (ThermoFisher Scientific). Reverse-transcription of 500ng RNA to cDNA was performed using qScript cDNA Supermix (VWR, 95048-100) according to manufacturer's instructions. qRT-PCR was performed using PerfeCTa FastMix II (VWR, 95118-012). Fluorescence real-time PCR reactions were run using the C100 Touch Thermal Cycler instrument (BioRad). All FAM-Labeled TaqMan Probes were normalized to endogenous control eukaryotic 18S ribosomal RNA (Thermo, 4319413E). The 2(2ddC(T)) method was used for quantitation of relative mRNA levels and fold induction.

Immunoblot Blot Assay

ISG15 antibody (21900-1) was purchased from PBL Assay Science. OCT4 (ab19857), UBE1L (ab133479), UBE2L6 (ab109086), and SOCS1 (ab280886) antibodies were purchased from Abcam. STAT1 (9172S) and pSTAT1 (9167) antibodies were purchased from Cell Signaling. AFP (A8452) and Albumin (A6684) antibodies were purchased from Millipore/Sigma. FOXA2 antibody (sc-374376) was purchased from Santa Cruz. Anti-Mouse IgG HRP-conjugate (W4021) and Anti-Rabbit IgG HRP-conjugate (W4011) antibodies were purchased from Promega. Intracellular protein was isolated from cells using 1x Laemmli Buffer (ThermoFisher Scientific, 34577) with addition of 5% BME (ThermoFisher Scientific, 21985023). Samples were processed following BioRad protocol; 10% or 7.5% sodium dodecyl sulfate-polyacrylamide gel electrophoresis (SDS-PAGE) followed by transfer onto polyvinylidene fluoride (PVDF) membrane. ECL

Super Signal solution was used (ThermoFisher Scientific, 34577) for chemiluminescence imaging using Blue X-Ray film (VWR, 101100-098) or ChemiDoc Imaging System (BioRad).

Immunoprecipitation (IP) Assay

HepaRG cells were grown to 60% confluence followed by treatment with or without interferon alpha (IFNα; 1000U/mL) for 24 hours. Following treatment, cells were lysed using RIPA Lysis Buffer System (Santa Cruz Biotechnology). Total protein concentrations in the lysate were calculated using Pierce Coomassie Protein Assay Kit (ThermoFisher Scientific) according to the manufacturer's protocol. 1ug of anti-ISG15 mouse monoclonal antibody (PBL Assay Science) or normal mouse IgG was incubated with prepared agarose resin; 50uL of protein A/G plus-agarose (Santa Cruz Biotechnology). 500ug of lysate was added relative to each condition and incubated overnight at 4°C with shaking. Samples were washed with 1x PBS and RIPA buffer. To elute the associated ISGylated proteins, laemmli sample buffer (BioRad) was diluted to 1x in PBS and 50uL was added to each IP. IPs were heated to 95°C for 10 min. Samples were analyzed by immunoblot probing for STAT1 and ISG15.

Reduced Representative Bisulfate Sequencing (RRBS)

RRBS assay for the *SOCS1* gene in iPSC and HepaRG cells was performed by Active Motif (Carlsbad, CA, USA). RRBS DNA Methylation assays was performed according to manufacturer's protocol. Genomic DNA was extracted using the Quick-gDNA MiniPrep kit (ZymoResearch, D3024). Following enzymatic digestion, bisulfite conversion was performed using the EpiTect Fast DNA Bisulfite Kit (Qiagen, 59824). Ovation RRBS Methyl-Seq System (Tecan 0353-32) was used for sample library preparation and quantified using the Agilent 2200 TapeStation System and the KAPA Library Quant Kit ABI Prism qPCR Mix (Roche

KK4835). The prepared libraries were sequenced on the NextSeq 550 at single end reads of 75 nt. RRBS data was mapped to the human genome GRCh37/hg19.

Chromatin Immunoprecipitation qPCR (ChIP qPCR)

HepaRG and iPSC cells were fixed using Formaldehyde buffer (37% Formaldehyde (Sigma, F-8775), 5M NaCl (Thermo, AM9760G), 0.5M EDTA (Sigma, 03690- 100mL) 1M HEPES (Thermo, 15630-080), and sterile water) for 15 min, quenched with 2.5 M glycine (Sigma, G-7403), treated with 0.05% Igepal detergent (Sigma, I-8896) in 1x PBS solution (ThermoFisher, 10010023) supplemented with the protease inhibitor phenylmethylsulfonyl fluoride (PMSF, Sigma, P-7626). Samples were sent to Active Motif for chromatin analysis. Chromatin was isolated by adding lysis buffer, followed by disruption with a Dounce homogenizer. Lysates were sonicated and the DNA sheared to an average length of 300-500 bp with Active Motif's EpiShear probe sonicator (cat# 53051). Genomic DNA (Input) was prepared by treating aliquots of chromatin with RNase, proteinase K and boiled for de-crosslinking, followed by SPRI beads clean up (Beckman Coulter) and quantitation using the Clariostar (BMG Labtech). An aliquot of chromatin (30 µg) was precleared with protein A agarose beads (ThermoFisher Scientific). Genomic DNA regions of interest were isolated using 4 µl of antibodies against H3K4me1, H3K4me3 and H3K27Ac (Active Motif, catalog numbers 39297, 39159 and 39133, respectively). Complexes were eluted from the beads, Crosslinks were reversed, and ChIP DNA was purified by phenol-chloroform extraction and ethanol precipitation. Ready-to-ChIP Chromatin and ChIP reactions were analyzed by qPCR. Primers used for ChIP-PCR were designed by Active Motif to human SOCS1 at three genomic regions on chromosome 16. Regions of interest for ChIP qPCR primers were mapped to human genome build GRCh37/hg19. Primer sequences for region 1 are: 5'-ATCCCTTGATGCTGGTTCAG-3' (forward) and 5'-AGAGGAAACTTCTTTGGTTCTATG-3' (reverse); region 2 are: 5'-ACACGGCATCCCAGTTAATG-3' (forward) and 5'-GTCCTCCGCGACTACCTGAG-3' (reverse);

region 3 are: 5’-CTCTGCTGCTGTGGAGACTG-3’ (forward) and 5’-GCCCCTTCTGTAGGATGGTAG-3’ (reverse). Quantitative PCR (qPCR) reactions were carried out in triplicate for specific genomic regions using SYBR Green Supermix (BioRad, Cat # 170-8882) on a CFX Connect™ Real Time PCR system. The resulting signals were normalized for primer efficiency by carrying out qPCR for each primer pair using input DNA from each cell types. A negative control primer set (Active Motif, 71001) was also used.

II. Supplementary figures

Fig. S1.

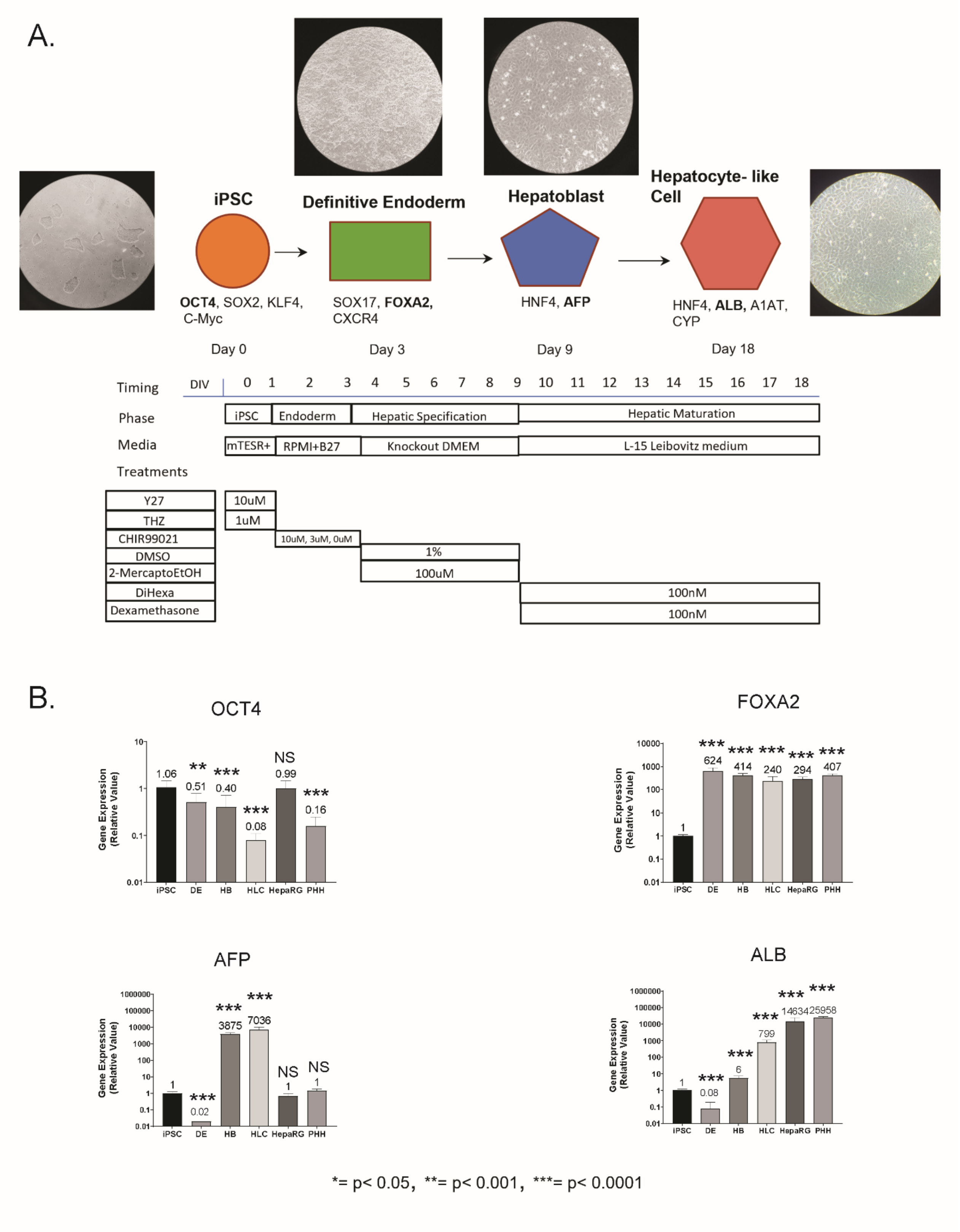

Fig. S1. Protocol for differentiation of induced-pluripotent stem cells (iPSC) to hepatocyte-like cells (HLC). (A) 18-Day protocol for differentiating iPSCs to HLCs using small molecules. Inverted microscope bright-field images are at 20x magnification for each major stage of differentiation. (B) qPCR analysis of mRNA expression for cell markers: OCT4 for iPSC, FOXA2 for DE, AFP for HB, and albumin (ALB) for HLCs. Samples were collected at the end of each stage: day 1 for iPSCs, day 4 for DE, day 10 for HB and day 18 for HLC. Data from repeated experiments in triplicate were averaged and are expressed as mean and standard deviation values (error bar) presented with an unpaired student's t-test with Welch's correction used to determine the p-values. A p-value <0.05 was considered significant. * = p< 0.05, **= p<0.001, ***= p<0.0001, NS=non-significant.

Fig. S2.

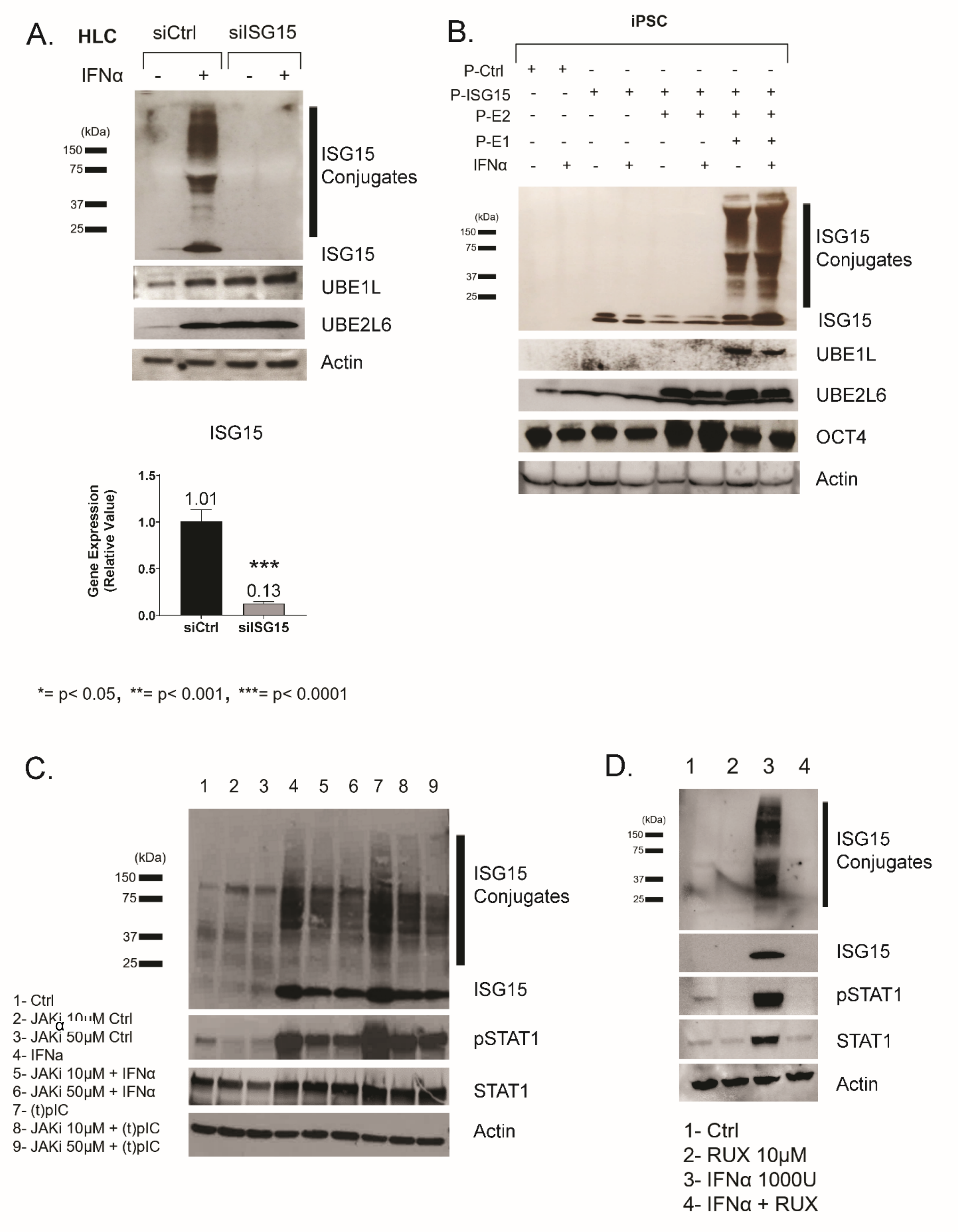

A.
HLC
siCtrl
siISG15
IFNα
(kDa)
150
75
37
25
ISG15 Conjugates
ISG15
UBE1L
UBE2L6
Actin
ISG15
Gene Expression (Relative Value)
1.01
***
0.13
siCtrl
siISG15
*= p< 0.05, **= p< 0.001, ***= p< 0.0001
B.
iPSC
P-Ctrl
P-ISG15
P-E2
P-E1
IFNα
ISG15 Conjugates
ISG15
UBE1L
UBE2L6
OCT4
Actin
C.
1 2 3 4 5 6 7 8 9
ISG15 Conjugates
ISG15
pSTAT1
STAT1
Actin
1- Ctrl
3- JAKi 50μM Ctrl
4- IFNa
5- JAKi 10μM + IFNα
6- JAKi 50μM + IFNα
7- (t)pIC
8- JAKi 10μM + (t)pIC
9- JAKi 50μM + (t)pIC
D.
1 2 3 4
ISG15 Conjugates
ISG15
pSTAT1
STAT1
Actin
1- Ctrl
2- RUX 10μM
3- IFNα 1000U
4- IFNα + RUX

Fig. S2. Mechanistic studies targeting ISGylation in HLCs and HepaRG cells. (A) Protein and mRNA expression analysis (qPCR) of ISG15 siRNA treated HLCs. Cells were treated with 50µM of ISG15 for 48 hours before the addition of 1000U/mL IFNα for an additional 24 hours. qPCR Data from repeated experiments in triplicate were averaged and are expressed as mean and standard deviation values (error bar) presented with an unpaired student's t-test with Welch's correction used to determine the p-values. A p-value <0.05 was considered significant. * = p< 0.05, **= p<0.001, ***= p<0.0001, NS=non-significant. (B) Western blot analysis of transfected gene expression plasmids for ISG15, UBE2L6 and UBE1L. IMR90 iPSCs were treated with 1µg of control plasmid, ISG15 plasmid, UBE1L plasmid and/or UBE2L6 plasmid for 24 hours simultaneously with 1000U/mL IFNα. (C-D) HepaRG cells were treated with 10 or 50µM of JAK1/2 inhibitor (JAKi) or 10 µM Ruxolitinib (RUX) for 5 hours prior to adding 1000U/mL of IFNα or 500ng of transfected poly(I:C)=(t)PIC for an additional 24 hours. (C) Western blot analysis of protein expression for ISG15, ISGylation, pSTAT1, STAT1 and β-actin following JAK1/2 inhibitor treatment. (D) Western blot analysis of protein expression for ISG15, ISGylation, pSTAT1, STAT1 and β-actin following treatment with Ruxolitinib.

Fig. S3.

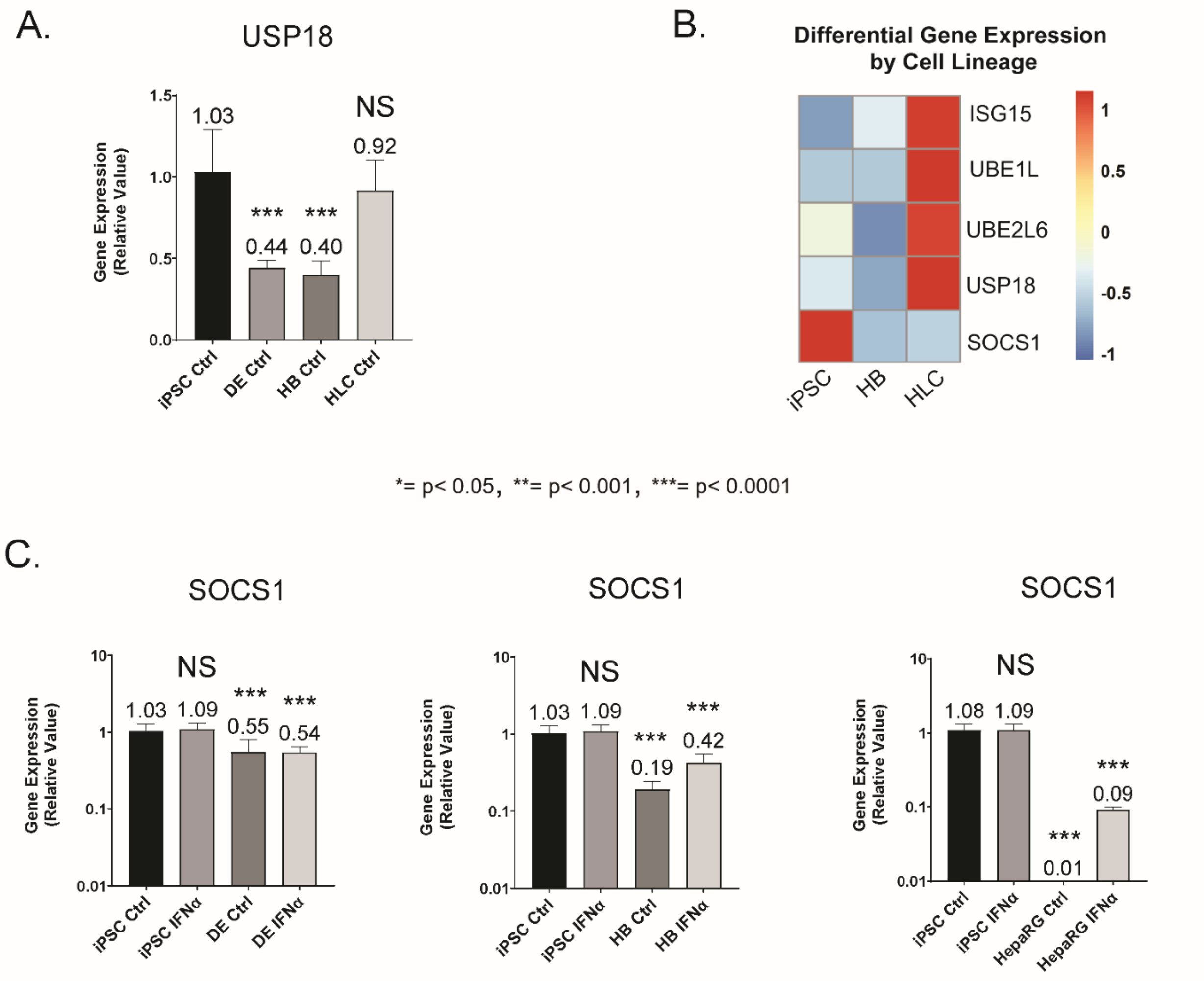
A.
USP18
Gene Expression (Relative Value)
1.03
0.44
0.40
0.92
NS
***
***
iPSC Ctrl
DE Ctrl
HB Ctrl
HLC Ctrl
B.
Differential Gene Expression by Cell Lineage
ISG15
UBE1L
UBE2L6
USP18
SOCS1
iPSC
HB
HLC
*= p< 0.05, **= p< 0.001, ***= p< 0.0001
C.
SOCS1
1.03
1.09
0.55
0.54
NS
***
***
iPSC Ctrl
iPSC IFNα
DE Ctrl
DE IFNα
SOCS1
1.03
1.09
0.19
0.42
NS
***
***
iPSC Ctrl
iPSC IFNα
HB Ctrl
HB IFNα
SOCS1
1.08
1.09
0.01
0.09
NS
***
***
iPSC Ctrl
iPSC IFNα
HepaRG Ctrl
HepaRG IFNα

Fig. S3. SOCS1 and USP18 protein and mRNA expression during iPSC differentiation to HLCs. (A) qPCR analysis of USP18 baseline expression in iPSCs, DEs, HBs and HLCs. Data from repeated experiments in triplicate were averaged and are expressed as mean and standard deviation values (error bar) presented with an unpaired student's t-test with Welch's correction used to determine the p-values. A p-value <0.05 was considered significant. * = p< 0.05, **= p<0.001, ***= p<0.0001, NS=non-significant. (B) RNA Seq heatmap of ISG15, UBE1L, UBE2L6, USP18, and SOCS1 in iPSCs, HBs and HLCs. Data from Geo Accession ID: GSE97987. (C) qPCR analysis of genes for SOCS1 following 8 hours of IFNα treatment in iPSCs, DEs, HBs and HepaRG cells. Data from repeated experiments in triplicate were averaged and are expressed as mean and standard deviation values (error bar) presented with an unpaired student's t-test with Welch's correction used to determine the p-values. A p-value <0.05 was considered significant. * = p< 0.05, **= p<0.001, ***= p<0.0001, NS=non-significant..

Fig. S4.

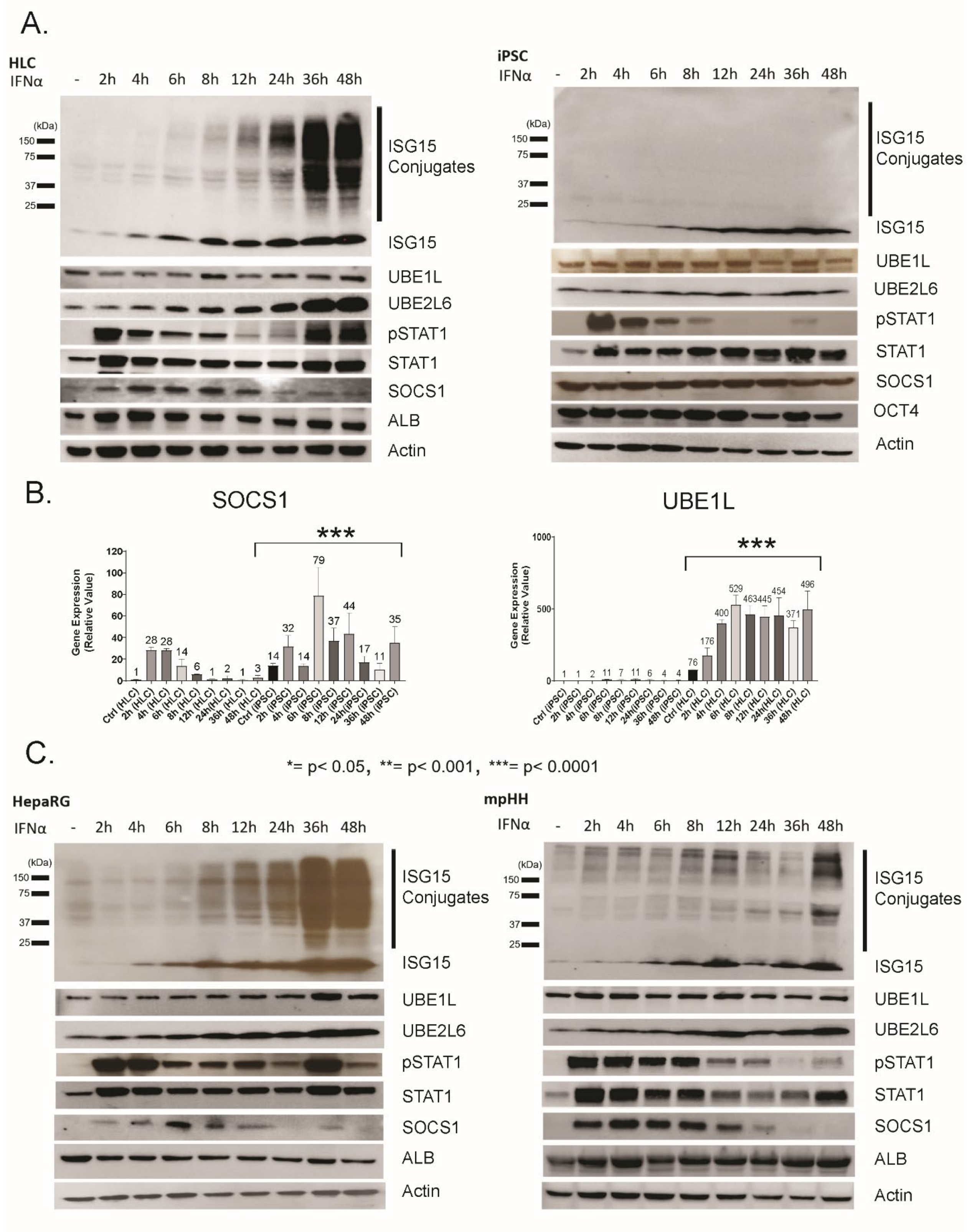
A.
HLC
IFNα - 2h 4h 6h 8h 12h 24h 36h 48h
(kDa)
150
75
37
25
ISG15 Conjugates
ISG15
UBE1L
UBE2L6
pSTAT1
STAT1
SOCS1
ALB
Actin
iPSC
IFNα - 2h 4h 6h 8h 12h 24h 36h 48h
(kDa)
150
75
37
25
ISG15 Conjugates
ISG15
UBE1L
UBE2L6
pSTAT1
STAT1
SOCS1
OCT4
Actin
B.
SOCS1
***
Gene Expression (Relative Value)
1 28 28 14 6 1 2 1 3 14 32 14 79 37 44 17 11 35
Ctrl (HLC) 2h (HLC) 4h (HLC) 6h (HLC) 8h (HLC) 12h (HLC) 24h(HLC) 36h (HLC) 48h (HLC) Ctrl (iPSC) 2h (iPSC) 4h (iPSC) 6h (iPSC) 8h (iPSC) 12h (iPSC) 24h(iPSC) 36h (iPSC) 48h (iPSC)
UBE1L
***
Gene Expression (Relative Value)
1 1 2 11 7 11 6 4 4 76 176 400 529 463 445 454 371 496
Ctrl (iPSC) 2h (iPSC) 4h (iPSC) 6h (iPSC) 8h (iPSC) 12h (iPSC) 24h(iPSC) 36h (iPSC) 48h (iPSC) Ctrl (HLC) 2h (HLC) 4h (HLC) 6h (HLC) 8h (HLC) 12h (HLC) 24h(HLC) 36h (HLC) 48h (HLC)
C.
*= p< 0.05, **= p< 0.001, ***= p< 0.0001
HepaRG
IFNα - 2h 4h 6h 8h 12h 24h 36h 48h
(kDa)
150
75
37
25
ISG15 Conjugates
ISG15
UBE1L
UBE2L6
pSTAT1
STAT1
SOCS1
ALB
Actin
mpHH
IFNα - 2h 4h 6h 8h 12h 24h 36h 48h
(kDa)
150
75
37
25
ISG15 Conjugates
ISG15
UBE1L
UBE2L6
pSTAT1
STAT1
SOCS1
ALB
Actin

Fig. S4. IFN stimulation time course in iPSCs, HLCs, HepaRG cells and PHHs. (A-C) Cells were treated with 1000U/mL of IFNα for indicated times (h=hours). (A and C) Western blot analysis of protein expression for ISG15, ISGylation, UBE1L, UBE2L6, pSTAT1, STAT1, β-actin, SOCS1, OCT4 and albumin (ALB). (B) qPCR analysis of mRNA expression for SOCS1 and UBE1L from iPSCs and HLCs. Data from repeated experiments in triplicate were averaged and are expressed as mean and standard deviation values (error bar) presented with an unpaired student's t-test with Welch's correction used to determine the p-values. A p-value <0.05 was considered significant. * = p< 0.05, **= p<0.001, ***= p<0.0001, NS=non-significant.

Fig. S5.

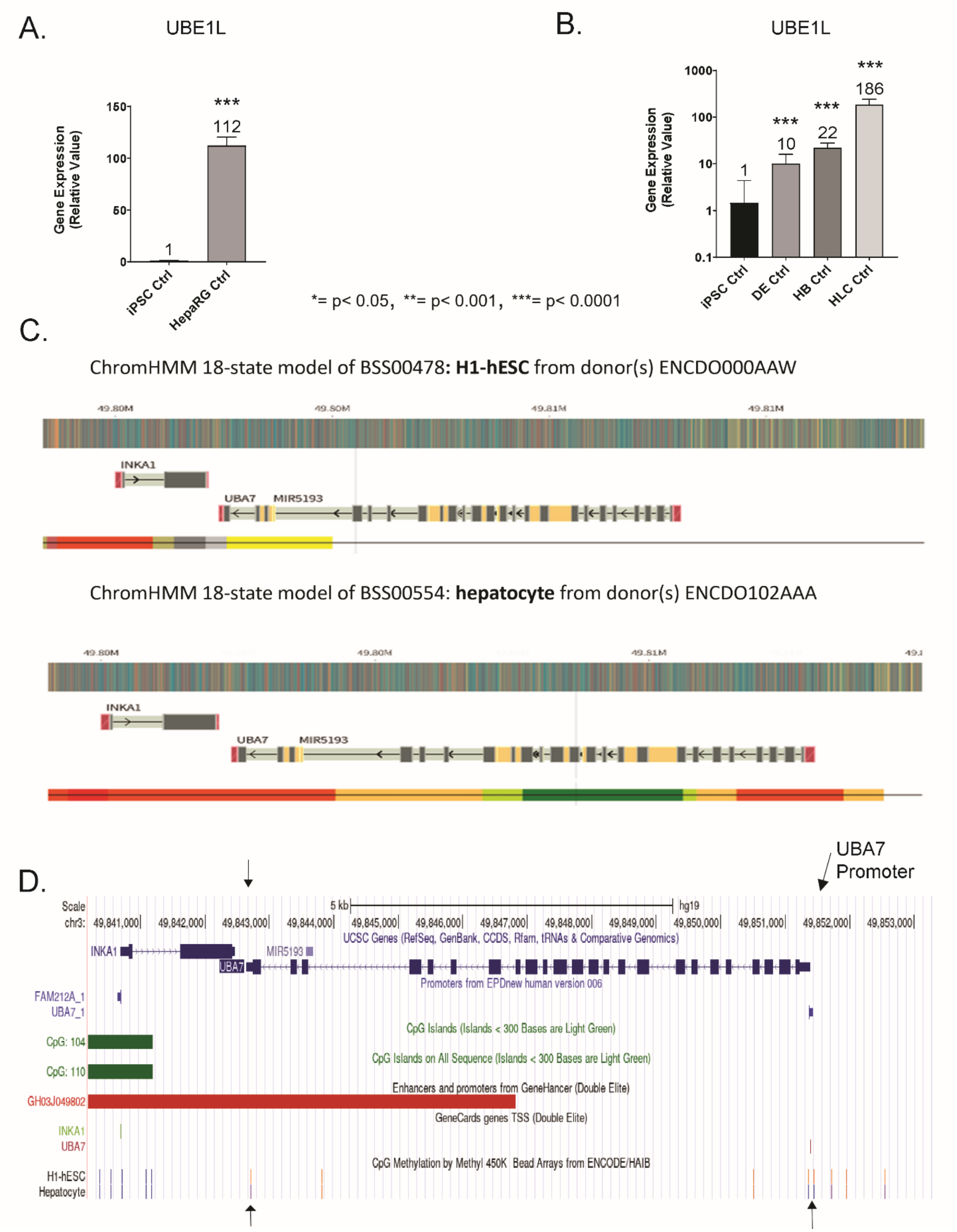
A.
UBE1L
Gene Expression (Relative Value)
150
100
50
0
***
112
1
iPSC Ctrl
HepaRG Ctrl
B.
UBE1L
Gene Expression (Relative Value)
1000
100
10
1
0.1
***
186
***
22
***
10
1
iPSC Ctrl
DE Ctrl
HB Ctrl
HLC Ctrl
*= p< 0.05, **= p< 0.001, ***= p< 0.0001
C.
ChromHMM 18-state model of BSS00478: H1-hESC from donor(s) ENCDO000AAW
INKA1
UBA7
MIR5193
ChromHMM 18-state model of BSS00554: hepatocyte from donor(s) ENCDO102AAA
INKA1
UBA7
MIR5193
D.
UBA7 Promoter
Scale
5 kb
hg19
chr3: 49,841,000 49,842,000 49,843,000 49,844,000 49,845,000 49,846,000 49,847,000 49,848,000 49,849,000 49,850,000 49,851,000 49,852,000 49,853,000
UCSC Genes (RefSeq, GenBank, CCDS, Rfam, tRNAs & Comparative Genomics)
INKA1
MIR5193
UBA7
Promoters from EPDnew human version 006
FAM212A_1
UBA7_1
CpG Islands (Islands < 300 Bases are Light Green)
CpG: 104
CpG Islands on All Sequence (Islands < 300 Bases are Light Green)
CpG: 110
Enhancers and promoters from GeneHancer (Double Elite)
GH03J049802
GeneCards genes TSS (Double Elite)
INKA1
UBA7
CpG Methylation by Methyl 450K Bead Arrays from ENCODE/HAIB
H1-hESC
Hepatocyte

Fig. S5. Regulation of baseline UBE1L expression. (A) qPCR analysis of baseline mRNA expression for UBE1L in iPSCs compared to HepaRG cells. Data from repeated experiments in triplicate were averaged and are expressed as mean and standard deviation values (error bar) presented with an unpaired student's t-test with Welch's correction used to determine the p-values. A p-value <0.05 was considered significant. * = p< 0.05, **= p<0.001, ***= p<0.0001, NS=non-significant. (B) qPCR analysis of baseline mRNA expression for UBE1L in iPSCs, DEs, HBs and HLCs. Data from repeated experiments in triplicate were averaged and are expressed as mean and standard deviation values (error bar) presented with an unpaired student's t-test with Welch's correction used to determine the p-values. A p-value <0.05 was considered significant. * = p< 0.05, **= p<0.001, ***= p<0.0001, NS=non-significant. (C) Chromatin regulatory regions in UBA7 (UBE1L) gene in human embryonic stem cells and primary hepatocytes from ENCODE database. (D) CpG methylation status of UBA7 (UBE1L) gene in human embryonic stem cells and human hepatocytes from UCSC Genome Browser. Orange = Methylated CpG site, Blue = Unmethylated CpG site, Purple = Partially Methylated CpG site.

https://genome.ucsc.edu/cgi-bin/hgTracks?db=hg19&lastVirtModeType=default&lastVirtModeExtraState=&virtModeType=default&virtMode=0&nonVirtPosition=&position=chr3%3A49840189%2D49853319&hgsid=1340921929_S7fTVBatgVyvqAslx5G3Nvf7mXaO

Fig. S6.

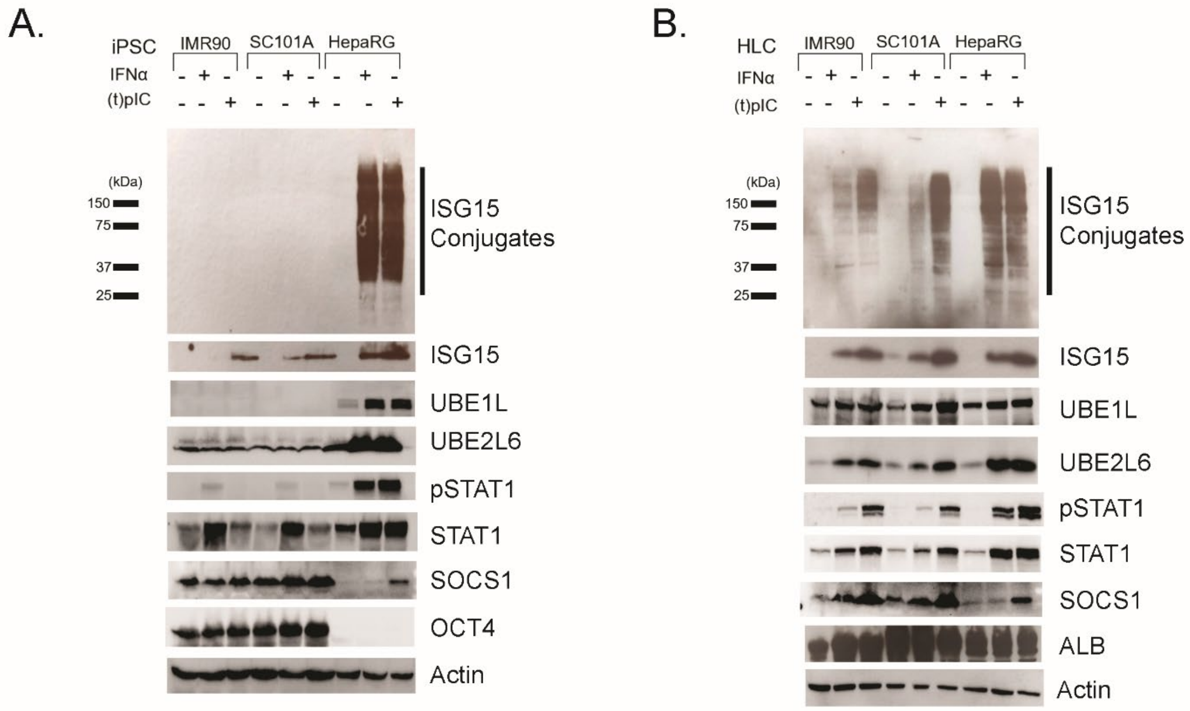
A.
iPSC
IMR90
SC101A
HepaRG
IFNα
(t)pIC
(kDa)
150
75
37
25
ISG15 Conjugates
ISG15
UBE1L
UBE2L6
pSTAT1
STAT1
SOCS1
OCT4
Actin
B.
HLC
IMR90
SC101A
HepaRG
IFNα
(t)pIC
(kDa)
150
75
37
25
ISG15 Conjugates
ISG15
UBE1L
UBE2L6
pSTAT1
STAT1
SOCS1
ALB
Actin

Fig. S6. ISGylation is not detectable in the IMR90 induced-pluripotent stem cell line at the iPSC stage. (A-B) Cells were treated with 1000U/mL IFNα or 500ng transfected poly(I:C) = (t)pIC for 24 hours. Protein expression for ISG15, UBE1L, UBE2L6, pSTAT1, STAT1, SOCS1, β-actin and cell markers OCT4 and ALB. (A) Western blot analysis of induced pluripotent stem cell lines (IMR90 and SC101A) and HepaRG cells. (B) Western blot analysis of hepatocyte-like cells (IMR90 and SC101A) and HepaRG cells.

III. Supplementary references

1. Ishida, Y., et al., *Novel robust in vitro hepatitis B virus infection model using fresh human hepatocytes isolated from humanized mice.* Am J Pathol, 2015. **185**(5): p. 1275-85.
2. Asumda, F.Z., et al., *Differentiation of hepatocyte-like cells from human pluripotent stem cells using small molecules.* Differentiation, 2018. **101**: p. 16-24.
3. Wakita, T., et al., *Production of infectious hepatitis C virus in tissue culture from a cloned viral genome.* Nat Med, 2005. **11**(7): p. 791-6.
