# Supplementary material for: Downregulation of SOCS1 increases interferon-induced ISGylation during differentiation of induced-pluripotent stem cells to hepatocytes
Source: JHEP Rep. 2022 Sep 23;4(12):100592. doi: 10.1016/j.jhepr.2022.100592 (PMC9685392; doi:10.1016/j.jhepr.2022.100592)
Supplement: Multimedia component 2 [file mmc2.docx]

**Journal of Hepatology**

**CTAT methods**

Tables for a “Complete, Transparent, Accurate and Timely account” (CTAT) are now mandatory for all revised submissions. The aim is to enhance the reproducibility of methods.

- Only include the parts relevant to your study
- Refer to the CTAT in the main text as ‘Supplementary CTAT Table’
- Do not add subheadings
- Add as many rows as needed to include all information
- Only include one item per row

**If the CTAT form is not relevant to your study, please outline the reasons why:**

|  |
| --- |

- 1. **Antibodies**

| **Name** | **Citation** | **Supplier** | **Cat no.** | **Clone no.** |
| --- | --- | --- | --- | --- |
| ISG15 antibody |  | PBL Assay Science | 21900-1 |  |
| UBE1L |  | Abcam | ab133479 |  |
| UBE2L6 |  | Abcam | ab109086 |  |
| SOCS1 |  | Abcam | ab280886 |  |
| STAT1 |  | Cell Signaling | 9172S |  |
| pSTAT1 |  | Cell Signaling | 9167 |  |
| AFP |  | Millipore/Sigma | A8452 |  |
| Albumin |  | Millipore/Sigma | A6684 |  |
| FOXA2 |  | Santa Cruz | sc-374376 |  |

- 1. **Cell lines**

| **Name** | **Citation** | **Supplier** | **Cat no.** | **Passage no.** | **Authentication test method** |
| --- | --- | --- | --- | --- | --- |
| **HepaRG** |  | ThermoFisher Scientific | **HPRGC10** |  |  |
| HepG2 cells |  | ATCC | HB-8065 |  |  |

- 1. **Organisms**

| **Name** | **Citation** | **Supplier** | **Strain** | **Sex** | **Age** | **Overall n number** |
| --- | --- | --- | --- | --- | --- | --- |
| **Not Applicable** |  |  |  |  |  |  |

- 1. **Sequence based reagents**

| **Name** | **Sequence** | **Supplier** |
| --- | --- | --- |
| ISG15, UBE1L, UBE2L6, STAT1, SOCS1, OCT4, FOXA2, AFP, and albumin | Available on the IDT Website | TaqMan Probes for quantitative Real Time PCR (qRT-PCR) analysis were purchased from Integrated DNA Technologies (IDT) |

- 1. **Biological samples**

| **Description** | **Source** | **Identifier** |
| --- | --- | --- |
| **Not Applicable** |  |  |

- 1. **Deposited data**

| **Name of repository** | **Identifier** | **Link** |
| --- | --- | --- |
| **Geo Database** | GSE211161 | https://www.ncbi.nlm.nih.gov/geo/query/acc.cgi?acc=GSE211161 |

- 1. **Software**

| **Software name** | **Manufacturer** | **Version** |
| --- | --- | --- |
| **Not Applicable** |  |  |

- 1. **Other (e.g. drugs, proteins, vectors etc.)**

| **Not Applicable** |  |  |
| --- | --- | --- |
|  |  |  |

- 1. **Please provide the details of the corresponding methods author for the manuscript:**

| **Emmanuel Thomas, University of Miami Miller School of Medicine, 1550 NW 10^th^ Ave, Rm. PAP 109, Miami, FL 33136, USA, Ph. +013052432895, email: ethomas1@med.miami.edu** |
| --- |

**2.0 Please confirm for randomised controlled trials all versions of the clinical protocol are included in the submission. These will be published online as supplementary information.**

| **Not Applicable** |
| --- |
